# Advances in the use of morphogenic regulators and peptide regenerating factors for boosting plant transformation and genome editing

**DOI:** 10.3389/fpls.2026.1699984

**Published:** 2026-03-13

**Authors:** V. Mohan Murali Achary, Easter D. Syombua, Simranjit Kaur, Sri Cindhuri Katamreddy, Danni Zou, Sarah J. Hearne, Anindya Bandyopadhyay

**Affiliations:** 1 International Maize and Wheat Improvement Center (CIMMYT), Genetic Resources Program, Mexico-Veracruz, Texcoco, Mexico; 2 Department of Biotechnology, TERI School of Advanced Studies, Vasant Kunj, New Delhi, India

**Keywords:** CRISPR, gene editing, morphogenic regulators, plant transformation, regeneration, tissue culture

## Abstract

Plant regeneration and transformation remain significant bottlenecks towards the genetic improvement of most crop species by either genome editing or transgenic approaches. Recent research has therefore, transitioned from manual optimization of culture media and components to the use of morphogenic regulators (MRs) and novel peptide regeneration factors that can reprogram somatic cell fate to a totipotent state. For instance, the co-expression of TRs such as *GROWTH-REGULATING FACTORs (GRF), and GRF-INTERACTING FACTOR* (*GIF*) have been shown to facilitate regeneration of transgenic plants from recalcitrant varieties. Genotype dependence and low regenerabilty have also constrained the adoption of precision breeding tools such as *Cas9*, *Cas12a*, *Cas13*, base and prime editors for trait improvement in some species and genotypes. This review first explores the status of plant transformation and gene editing techniques, then discusses the mechanisms of key TRs, including those from the *WOX*, *DOF*, *AP2/ERF*, *LRR-RLK* and *KNOX* families, and emerging peptide factors like REF1. The review further outlines strategies to deploy these factors via constitutive, tissue-specific, transient, or inducible expression, and highlights how they expedite the production of transgenic and edited events. We have also reviewed applications across monocots (maize, rice, wheat, sorghum), dicots (soybean, rapeseed, tomato, sugar beet), and recalcitrant species (cassava, cacao, tree crops, medicinal plants). We further discuss challenges such as abnormal phenotypes and regulatory hurdles, and survey recent innovations, including inducible CRISPR activation of endogenous genes and new regeneration peptides that pave the way toward more efficient, genotype-flexible plant transformation and gene editing. Overall, this review seeks to highlight recent advancements and future perspectives in the application of TRs and peptide regenerating factors to overcome limitations in advanced biotechnological approaches, hence enhance plant resilience and productivity.

## 1 Introduction

The development of new crop varieties with enhanced traits is a crucial strategy for addressing the increasing demand for food and feed amidst climate change, biodiversity loss, declining arable land, and increased incidence of agricultural pests and diseases (Muluneh, 2021). Since marketing of the first transgenic crop in 1994 and most recently, the gene editing technology, biotech has significantly enhanced food security and catalyzed economic advancement by facilitating gene modification to incorporate desirable traits (Tripathi et al., 2022). Notably, traditional methods for *in vitro* transformation hinge on delivery of DNA or genome-editing reagents into explants, followed by extensive tissue culture to regenerate whole plants. These processes are time-consuming, highly genotype-dependent, and prone to somaclonal variation, thus quite inefficient (Maher et al., 2020). For example, although standard methods for *Agro*-mediated transformation have worked in several model crops, and select monocots and dicots, they’re only applicable in a narrow range of cultivars. Besides, they often demand laborious media optimization and stringent selection regimes to obtain transformed events. The second most-common transformation system, particle bombardment, often causes chromosomal rearrangements, cell damage and often integrates in multiple copies, and has the pre-requisite of a regenerable target tissue. On the other side, PEG-based DNA delivery to protoplasts has only been demonstrated in a few crop species (Brandizzi et al., 2025; Zhong et al., 2024). The effective development of stably transformed plants, therefore, remains a substantial bottleneck in most agriculturally important crops (Lee and Wang, 2023).

Recent studies have uncovered a distinct set of peptide regenerating factors and plant morphoregulatory genes (MRs) that actively facilitate plant regeneration. Further research demonstrated that the ectopic expression or targeted regulation of these genes improves the efficiency of plant transformation by unlocking the regenerative ability of somatic cells in previously unresponsive tissues (Debernardi et al., 2020; Hoerster et al., 2020; Jorge et al., 2021; Lowe et al., 2018; Maren et al., 2022; Xu et al., 2022; Yang et al., 2024; Lee and Wang, 2023). Characterization of the molecular mechanism of MRs has shown that they govern the biosynthetic and signaling pathways of auxins and cytokinins, thereby regulate plant regeneration processes (Gordon-Kamm et al., 2019).

According to (Maren et al., 2022), MRs encode transcription factors or signaling molecules that control cellular differentiation, embryogenesis, organogenesis, meristem identity and the overall plant regeneration process (Maren et al., 2022). For instance, the landmark study by (Lowe et al., 2016) found that the constitutive overexpression of the MRs *BABY BOOM* (*BBM*, an AP2/ERF-family transcription factor) and *WUSCHEL2* (*WUS2*, a homeodomain transcription factor) improves maize transformation efficiencies. The MRs enables transformation of maize multiple inbred lines that were previously considered recalcitrant, with up to twenty-fold increase in shoot regeneration and highly reduced genotype specificity. The researchers reported that co-expression of *BBM* and *WUS* keeps transformed cells in a dedifferentiated, embryogenic state and promotes rapid somatic embryo formation, effectively bypassing a prolonged callus phase. This MR combination also expands the range of ‘regenerable’ and ‘transformable’ explants, including maize mature seeds (Lowe et al., 2016) and leaf bases of rice, pearl millet, maize and sorghum (Wang et al., 2023). Instead of solely relying on synthetic hormone regimes, plant transformation systems based on MRs benefit from intrinsic embryogenesis pathways by globally resetting hormonal and developmental cues in the cell. For instance, *WUS* and *BBM* upregulate *LEAFY COTYLEDON1/2* (*LEC1/2*) and *AGL15*, which are master embryogenesis genes that modulate cytokinin and auxin pathways (Horstman et al., 2017). These insights indicate that an optimal combination of MRs can switch the balance from a wound response that typically results in cell death to a regeneration response to form somatic embryos and/or shoots. While *BBM* and *WUS* remain the core MRs, several other transcription factors and peptide regenerating factors, such as regeneration factor 1 (REF1), *GRF*-*GIF* chimeras, *WIND1*, and the *WOX* and *PLT5* gene families have been shown to exert synergistic effects on plant regeneration (Debernardi et al., 2020; Lee and Wang, 2023; Maher et al., 2020). By illuminating the molecular underpinnings of plant regeneration and cellular totipotency, our overarching objective is to advance precision breeding technologies through plant genetic transformation from diverse vantage points.

This review provides a comprehensive overview of how MRs and peptide factors are expediting plant transformation and gene editing. We first summarize the prevailing plant transformation techniques and recent advances in genome editing to set the context of the challenges these innovations aim to solve. In Section 3, we define morphogenic regulators and detail the mechanism of action and key examples then Section 4 discusses practical strategies for deploying these regulators in transformation and editing workflows. Sections 8 and 9 have addressed the challenges and limitations of these approaches and highlight recent advances and future prospects, including emerging peptide regeneration factors like REF1 and novel tools like inducible CRISPR activation of endogenous development genes.

## 2 Advancement in gene editing technology in basic and translational research

Engineered sequence-specific nucleases (SSNs), which are designed to identify and modify specific regions of a genome and introduce *in vivo* DNA breaks, are the main tool used in genome editing. For precise plant genome engineering, three key genome editing technologies have been employed: zinc finger nuclease (ZFN), transcription activator-like effector nuclease (TALEN), and clustered regularly interspaced short palindromic repeat (CRISPR)/CRISPR-associated protein 9 nuclease (Cas9). ZFNs and TALENs, which define target specificity through protein–DNA interactions, are costly and challenging to design. In contrast, CRISPR systems utilize RNA–DNA interactions to direct DNA targeting and cleavage, and are simple, highly efficient, and cost-effective. In recent years, CRISPR systems have become the most popular genome editing technology and are used widely across a variety of plant species (Zhu et al., 2020; Hassan et al., 2021). Since the CRISPR/Cas9 and CRISPR/Cpf1 systems offer simplicity of manipulation and targeted mutagenesis, they have been widely used in different fields. The various CRISPR reagents and new advancements that have gradually expanded the usefulness and efficiency of genome editing techniques in plants are covered in this section.

The CRISPR-Cas type-II nucleases have become widely used in genome editing research since they were discovered in *Streptococcus pyogenes* (*SpCas9*). For targeting DNA, it forms a complex with a single guide RNA (sgRNA), and for DNA recognition The Cas9-sgRNA complex attaches to the target sequence after Cas9 recognizes its PAM sequence, creating a double strand DNA break (DSB) at the target location by recognition domain (RuvC) and the nuclease domains (HNH) of the Cas9 protein. *Staphylococcus aureus* Cas9 (SaCa9) is a prominent and naturally occurring variation of these. High target specificity and low off-target Cas variants have been used for plant genome editing including *eSpCas9* (Zhang et al., 2019a), SpCas9-HF1 (Zhang et al., 2019a; Xu et al., 2019), HypaCas9 (Xu et al., 2019), eHF1-Cas9 (Liang et al., 2018), eHypa-Cas9 (Liang et al., 2018), HiFi Cas9 (Banakar et al., 2020), SaCas9 (Wolter et al., 2018) and xCas9 (Wang et al., 2019);. Similarly, alternate PAM has been developed and used for genome editing in various crop species including SpCas9-VQR (Hu et al., 2016), SpCas9-EQR (Yamamoto et al., 2019), SpCas9-VRER (Hu et al., 2016) and St1Cas9 (Steinert et al., 2015). Flexible PAM variants including SpCas9-NG (Qin et al., 2020), SaCas9-KKH (Qin et al., 2019), ScCas9 (Wang et al., 2020a), XNG-Cas9 (Niu et al., 2020), SpRY (Li et al., 2021a), SpCas9-NRRH (Li et al., 2021a), SpCas9-NRCH (Li et al., 2021a) and SpCas9-NRTH (Li et al., 2021a).

Cas12 nucleases are the second most used Cas proteins in plants belonging to class 2 type V CRISPR system consists of two main components, crRNA and Cpf1 nuclease. Cpf1 only needs one short CRISPR RNA (crRNA, about 42 nt), is a low-molecular-weight multifunctional effector protein that is not only involved in targeted DNA cleavage, but also in the processing of pre-crRNA (Fonfara et al., 2016). Various natural variants of Cas12 have been utilized in genome editing studies, including AsCas12a (Malzahn et al., 2019; Bernabé-Orts et al., 2019), LbCas12a (Malzahn et al., 2019; Schindele and Puchta, 2020), FnCas12a (Zhang et al., 2021a), AacCas12b (Ming et al., 2020), AaCas12b (Ming et al., 2020), BthCas12b (Ming et al., 2020), BhCas12b v4 (Wu et al., 2020a), BvCas12b (Wu et al., 2020a), Lb5Cas12a (Zhang et al., 2021a), BsCas12a (Zhang et al., 2021a), and Mb2Cas12a (Zhang et al., 2021a). Engineered variants of Cas12a, including LbCas12a-RR (Li et al., 2018), bCas12a-RVR (Li et al., 2018), and FnCas12a-RVR (Zhong et al., 2018), have been investigated in plants to broaden the PAM recognition sequence. Additionally, the temperature tolerance variant of Cas12a has also been demonstrated in plants, namely enLbCas12a (Schindele and Puchta, 2020), ttLbCas12a (Huang et al., 2021) and AacCas12b (Ming et al., 2020).

**Figure 1:**
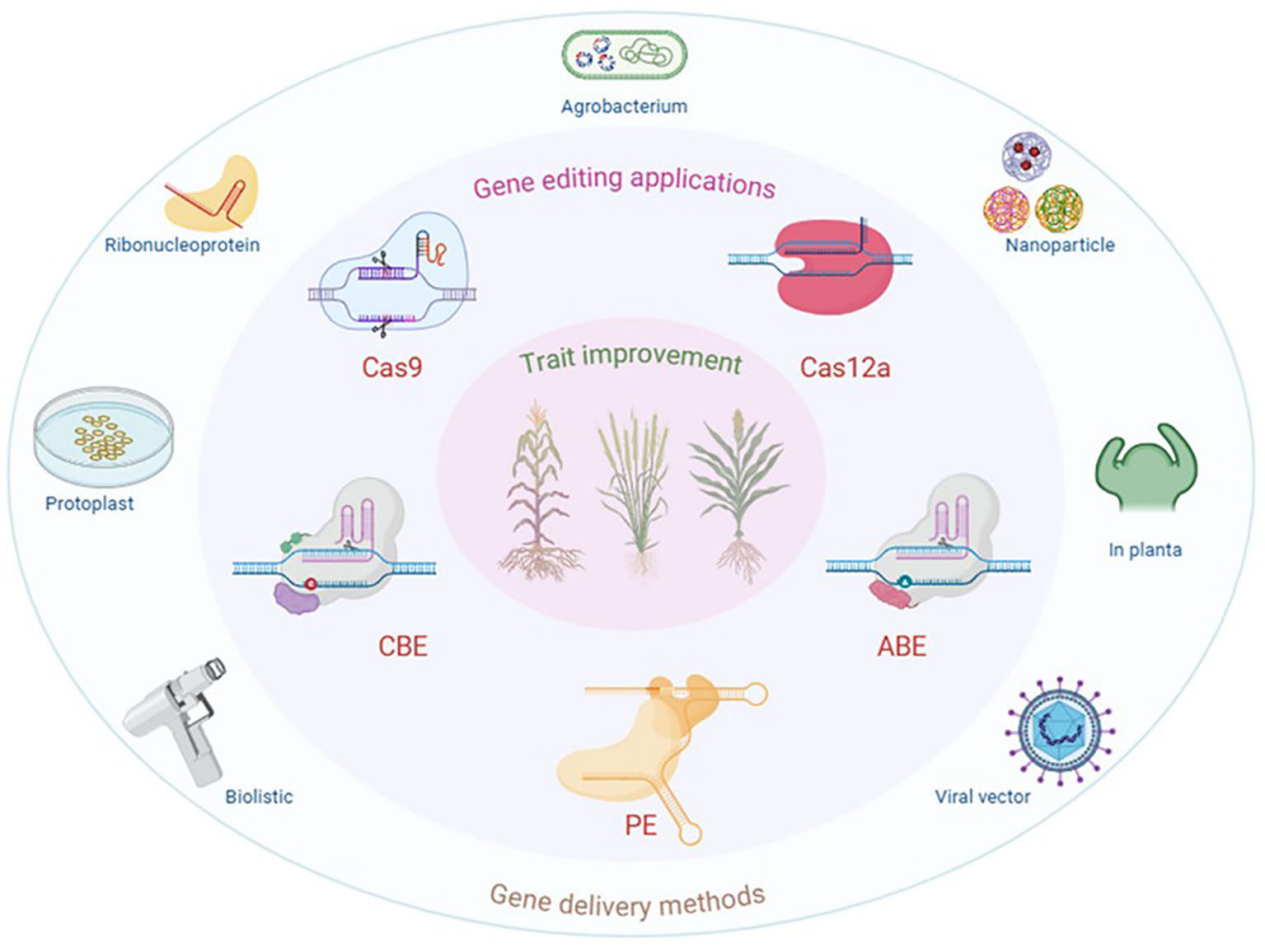
Applications of genome editing technology for trait improvement in plants. Methods for delivering CRISPR–Cas into plant cells include *Agrobacterium*-mediated genetic transformation to produce genome-edited plants. Other approaches involve the use of DNA plasmids or preassembled ribonucleoprotein complexes for protoplast transfection, viral vectors that carry gRNAs and Cas nucleases in either wild-type or Cas9-expressing plants, the biolistic method for introducing editing components into plant cells, and nanoparticles for mediating the delivery of DNA constructs for CRISPR–Cas expression into plant cells. Created in BioRender (2026). https://BioRender.com/dd7rsiq.

The base editor can facilitate precise base changes without introducing double stranded breaks (DSBs) or the requirement of additional donor DNA templates. The base editing system comprises two variants: cytosine base editor (CBE) and adenine base editor (ABE). These base editors use a combination of a base-modifying enzyme (deaminase) with a catalytically inactive Cas9 (dCas9) or nickase (nCas9) capable of only cleaving one strand (**Figure 1**). The nCas9 is typically used in modern base editors due to its superior performance over dCas9-based base editors. The CBE comprises the Cas9 nickase (D10A) conjugated with a cytidine deaminase (APOBEC1) and an inhibitor of uracil DNA glycosylase (UGI), facilitating the direct conversion of C:G to T:A at DNA target site, upstream of the PAM region (Komor et al., 2017). The ABE consists of nCas9 (D10A) coupled to adenosine deaminases, enabling the direct substitution of A:T with G:C by base pairing (Gaudelli et al., 2017). A dual base editor has been reported that effectively modifies C:G to T:A and A:T to G:C simultaneously (Li et al., 2020). CGBEs (C to G base editors) represent a novel category of base editors that can facilitate the transversion of C:G to G:C within a DNA molecule (Chen et al., 2021; Kurt et al., 2021; Zhao et al., 2021). The BE3 is the most efficient and has been extensively utilized in various plant species (Mishra et al., 2020; Gürel et al., 2019). Recently, PmCDA1-CBE_V04 and A3A/Y130FCBE_V04 exhibited high editing activity and specificity while minimizing indel byproducts (Ren et al., 2021). Further, TadA8e and TadA9 are ABEs recommended for converting A/T to G/C with extensive sequence compatibility and enhanced performance (Ren et al., 2021; Yan et al., 2021).

Currently, six out of the twelve possible base alterations can be accomplished using the existing base editors. In contrast, the prime editing system facilitates all twelve categories of base substitutions, with specific and accurate insertions and deletions, in addition to base transversions. The “prime editing tool” consists of an nCas9 (H840A) fusion enzyme coupled with a reverse transcriptase (rt) guided by a prime editor guide RNA (pegRNA). PegRNA consists of two primary components: a 20 nucleotide sgRNA sequence and a reverse transcription template that features a primer binding site (PBS) containing the desired mutation information. Several versions of prime editors have been developed over the years, including Prime Editor 1 (Anzalone et al., 2019), Prime Editor 2 (Anzalone et al., 2019), Prime Editor 3 (Xu et al., 2022), Prime Editor 4max (Vu et al., 2024), Prime Editor 6c (Cao et al., 2024), pH-ePPE (Zong et al., 2022), pH-nCas9-PPE-V2 (Lin et al., 2020) and ePPEplus (Ni et al., 2023). Several different crop species have successfully been edited by prime editing, including rice (Lin et al., 2020; Xu et al., 2020; Cao et al., 2024), maize (Qiao et al., 2023), wheat (Ni et al., 2023; Lin et al., 2021), potato (Perroud et al., 2022), and tomato (Vu et al., 2024). As advances in the field continue, genome editing is gaining advantages for an increasing number of crops and traits. Crop genome modification using CRISPR technology requires several crucial components, including the availability of genome sequences, a validated target gene, an effective DNA delivery system, suitable expression systems, and an improved tissue culture regeneration system.

## 3 Method of delivery of editing components into plant tissue

Across multiple kingdoms of life, CRISPR/Cas genome editing has been proven to be a powerful platform for editing the genome. The difficulties in delivering CRISPR/Cas editing tools to plant cells and in recovering plants with transmissible variations bring significant technological barriers for the CRISPR/Cas utilization in plants. The introduction of genome editing reagents into intact plant tissues has been achieved mainly through *Agrobacterium* and particle bombardment. However, the growing trend of alternative innovative approaches, such as *Agrobacterium*-mediated viral-based replicons, direct introduction of genome editing components as ribonucleoprotein complexes, and nanoparticles, is gaining significant attention. The next section illustrates these broad topics with specific examples. *Agrobacterium*-mediated plant transformation has been continuously improved since its discovery (Barton et al., 1983). Additionally, other alternative methods have been discussed for delivering editing components to the plant cell for efficient genome editing.

### 3.1 Enhanced *Agrobacterium*- mediated gene delivery using virulent plasmids

The development of T-DNA binary vector systems (Hoekema et al., 1983) and addition of several non-oncogenic *Agrobacterium* strains such as LBA4404 (Ooms et al., 1981), AGL1 (Lazo et al., 1991), and EHA strains 101 and 105 (Hood et al., 1993) greatly revolutionized the use of *Agrobacterium* in plant biotechnology. However, alternative methods are still needed for plants that are recalcitrant to *Agrobacterium* transformation. Strategies such as altering the expression of virulence genes and adding copies of various *Vir* genes to create ‘supervirulent’ strains and ‘superbinary vectors’ (pSB1) have, therefore, increased transformation frequencies in many plants (Komari, 1990; Hiei et al., 1994; Ishida et al., 1996). The ternary pVir system, described by Anand et al. (2018), represents a simplified and improved second-generation superbinary vector. Specifically, it was developed through modifications and corrections of the original vector (pSB1), which further enhanced transformation frequency (Anand et al., 2018). Additionally, pSB1 was further modified to incorporate an improved gentamicin selectable agent to minimize the spontaneous development of resistance to tetracycline. The resulting pVir system was not only smaller and more virulent than pSB1 but also failed to confer spontaneous to tetracycline resistance (Anand et al., 2018). Such modifications have streamlined and enhanced genetic transformation in elite maize and sorghum varieties that were historically recalcitrant to *Agrobacterium*-mediated transformation (Anand et al., 2018; Che et al., 2018). This resulted in improved transformation frequencies (31.1%) and single-copy integrations compared to pSB1 (13.7%). Indeed, transferring the vir genes to a separate plasmid drastically reduced complexity and further streamlined the T-DNA vector construction. Furthermore, these systems have been used successfully with genome editing tools for editing purposes in various crop systems, leading to high editing efficiency (Wang et al., 2023). Helper plasmids that contain additional copies of key *Agrobacterium* virulence genes have been effectively utilized in various crops to enhance transformation efficiency (Anand et al., 2018; Zhang et al., 2019b; Kang et al., 2022a; Jeong et al., 2024; Aliu et al., 2024; Vandeputte et al., 2024). Additionally, the Type III Secretion System and its effector proteins from *Pseudomonas syringae* to supress plant immune response (Raman et al., 2022), as well as centrifugation (Ye et al., 2023) and sonication (Zhong et al., 2024) methods, have been used to enhance *Agrobacterium*-mediated transformation.

### 3.2 Biolistic-mediated gene delivery

The biolistic method is an alternative approach employed by researchers to introduce gene editing tools into plant systems that have historically proven resistant to *Agrobacterium*-mediated transformation. Once inside the cell, the microcarriers (tungsten or gold or silicon) coated with cargo biomolecules can transform not only the nucleus but also plastids and mitochondria, either by integrating the cargo into the genome or remaining as extrachromosomal material (Sanford, 2000). This system has many advantages, including the ability to target different tissue types and deliver large or multiple genome editing components on separate plasmids. However, the success of biolistic experiments is critically dependent on numerous parameters, including the acceleration of particles, target tissue types, microparticles size, and DNA to particles ratio, all of which must be carefully optimized to achieve efficient and precise genome editing. Biolistic delivery has been successfully employed for genome modification in a wide range of plant species, including wheat (Liang et al., 2017; Hamada et al., 2018) maize (Ainley et al., 2013a; Svitashev et al., 2015, Svitashev et al., 2016; Shi et al., 2017), rice (Sun et al., 2016) and soybean (Jacobs et al., 2015; Li et al., 2015). Recently, the flow guiding barrel (FGB) biolistic delivery method improved transient transfection efficiency by 22 times, CRISPR-Cas9 ribonucleoprotein editing efficiency by 4.5 times in onion epidermis, and viral infection efficiency by 17 times in maize seedlings. Furthermore, utilizing B104 immature embryos boosts stable transformation frequency in maize by more than tenfold and doubles the efficacy of CRISPR-Cas12a-mediated genome editing in wheat meristem tissue (Thorpe et al., 2025). The main disadvantages of this method include limited control over the distribution and penetration of microparticles, cell lethality, chromosome damage, and complex and multicopy vector DNA integration.

### 3.3 Virus vector-mediated gene delivery

Viruses are obligate parasites that unequivocally complete their genome replication within the host plant cells, and they frequently transmit between cells. Plant viruses-based replicons are being increasingly used as an alternative to *Agrobacterium* in the delivery of genome editing tools into plants, due to their ability to introduce the editing tools into plants at high levels of efficiency. The common viral components which have been replaced with editing reagents include the removal of viral coat proteins (Cody et al., 2017), movement proteins (Baltes et al., 2014), or vector-assisted transmission protein (Liu et al., 2023a). In the meantime, it will be difficult to employ such vectors for systemic or heritable modifications *in situ* as partially breaking down the virus genome may stop cell to cell movement. Tobacco rattle virus (TRV) possesses a broad host range and can invade to infect germline cells, making it one of the most frequently used viral vectorsand used in gene editing by multiple research groups in various plant species, including tobacco (Ellison et al., 2020) and *Arabidopsis* (Ghoshal et al., 2020; Nagalakshmi et al., 2022; Liu et al., 2022). Barley stripe mosaic virus (BYSMV), a positive strand RNA virus that infects many gramineae species, has been used to deliver editing reagents in various crops, including wheat (Hu et al., 2019; Li et al., 2021b; Wang et al., 2022a), maize (Hu et al., 2019), and barley (Tamilselvan-Nattar-Amutha et al., 2023). Similarly, Barley yellow striate mosaic virus (BYSMV) vector was used to deliver Cas9, and guide RNA in tobacco (Gao et al., 2019). In a similar vein, various positive RNA viruses have been utilized for genome editing, such as foxtail mosaic virus (FoMV) (Beernink et al., 2022; Mei et al., 2019), cotton leaf crumple virus (CLCrV) (Lei et al., 2022), tomato mosaic virus (ToMV) (Kaya et al., 2017), beet necrotic yellow vein virus (BNYVV) (Jiang et al., 2019), tobacco mosaic virus (Cody et al., 2017), and potato virus X (PVX) (Uranga et al., 2021).

### 3.4 Ribonucleoproteins -mediated gene delivery

*Agrobacterium*-mediated transformation or particle bombardment introduces CRISPR vector reagents into plant cells and tissues, facing regulatory obstacles due to random integration. Segregation eliminates transgenes, but it takes time and effort, making it untrainable for trees and vegetative plants. Genomic DNA is continuously exposed to CRISPR reagents during editing, inducing off-target mutations and chimeric mutants (Hashimoto et al., 2016). Plasmid-based CRISPR genome editing requires species-specific promoters, terminators, and codon modifications. Polyploid organisms may need several gRNAs, making CRISPR vector systems challenging. To address the challenges, the Cas protein and gRNAs can be preassembled into ribonucleoproteins (RNPs) for plant delivery to generate transgene-free events with reduced off-target effects (Kim et al., 2017; Liang et al., 2017). The efficiency of delivery plays a significant role in the performance of RNP-mediated editing. RNPs have previously been introduced into plants using four different techniques: lipofection, particle bombardment, electroporation, and cell transfection mediated by polyethylene glycol (PEG). Lipofection is regarded as a simple and affordable cell transfection technique (Liu et al., 2020). Another popular method for delivering RNPs into plant tissues and cells is particle bombardment. The RNP-coated particles can target explants that are commonly used for plant regeneration, such as leaf discs and embryos, with or without selection, and the explants can then be used for regeneration. In maize (Svitashev et al., 2016), wheat (Liang et al., 2017), and rice (Banakar et al., 2020), RNP-mediated genome editing through particle bombardment has been effectively demonstrated with editing efficiency less than 10% without any selection. Plasmids encoding a selective marker gene can be co-transformed into plants with RNPs to increase editing efficiency (Svitashev et al., 2016; Banakar et al., 2020). The most popular RNP-mediated delivery method is polyethylene glycol (PEG)-mediated cell transfection, which necessitates the pre-removal of the cell wall by pectinase and cellulase enzymes. The method allows for rapid evaluation of Cas systems in plant cells, and editing efficiencies can be determined between one and three days after transfection. PEG-mediated genome editing using protoplast assays have been used for many plant species including tobacco (Woo et al., 2015; Kim et al., 2017), maize (Sant’Ana et al., 2020), wheat (Liang et al., 2017), potato (Andersson et al., 2018; González et al., 2020), petunia (Yu et al., 2020), soybean (Kim and Choi, 2020), cabbage (Murovec et al., 2018), banana (Wu et al., 2020b) and rice, *Arabidopsis* and lettuce (Woo et al., 2015). To date, researchers have successfully edited entire plants from protoplasts in a variety of species, including rice (Barrera et al., 2025) and maize (Svitashev et al., 2016), potato (Moon et al., 2022) and wheat (Poddar et al., 2023). The rapid development of CRISPR and other biotechnologies makes RNP-mediated genetic engineering a promising platform for crop enhancement, broadening its applications in agriculture, food production, and research.

### 3.5 Challenges associated with plant transformation and regeneration

The success of discovery and translational research relies on the efficiency of plant transformation, which involves three critical steps: selection of appropriate explants, adoption of effective gene delivery systems, and regeneration of the complete plant from transformed cells (Lee and Wang, 2023; Syombua et al., 2021). Additional challenges encompass the restricted availability of explants, inadequate gene delivery systems, and the accessibility of transformation protocols to a limited number of species with restricted genotypes. Resistance to genetic transformation may arise from inadequate gene delivery to regenerative target cells and the limited regeneration capacity of explant material (Bidabadi and Jain, 2020; Long et al., 2022). It is often difficult for small laboratories to adopt established protocols as they require experienced technical personnel and the availability of high-quality explants (immature embryo) round the year. The outcome of plant transformation is largely dependent on cellular totipotency, which refers to the capacity of differentiated cells to dedifferentiate, attain embryonic stem cell characteristics, and regenerate into full-grown plants (Maren et al., 2022). Unlike some plant cells, not all plant cells are capable of undergoing transformation. For example, most monocot transformations utilize embryos or embryogenic calli as explants, whereas dicots use young leaves, leaf nodes, embryonic axes, shoot meristems, and leaf bases as explants (Chen et al., 2022a; Bélanger et al., 2024). The regeneration capacity of various explants exhibits significant variability among different genotypes, resulting in the regeneration of only a limited number of genotypes, which often do not include commercially and cultivated varieties, employing existing tissue culture protocols (Kausch et al., 2021; Long et al., 2022). The following sections summarize recent applications of MRs in transformation systems, as well as newly identified plant regeneration enhancers, signal peptides, and their potential role in improving transformation efficiency.

## 4 Key morphogenic regulators in plant transformation, regeneration and gene editing applications

**Figure 2:**
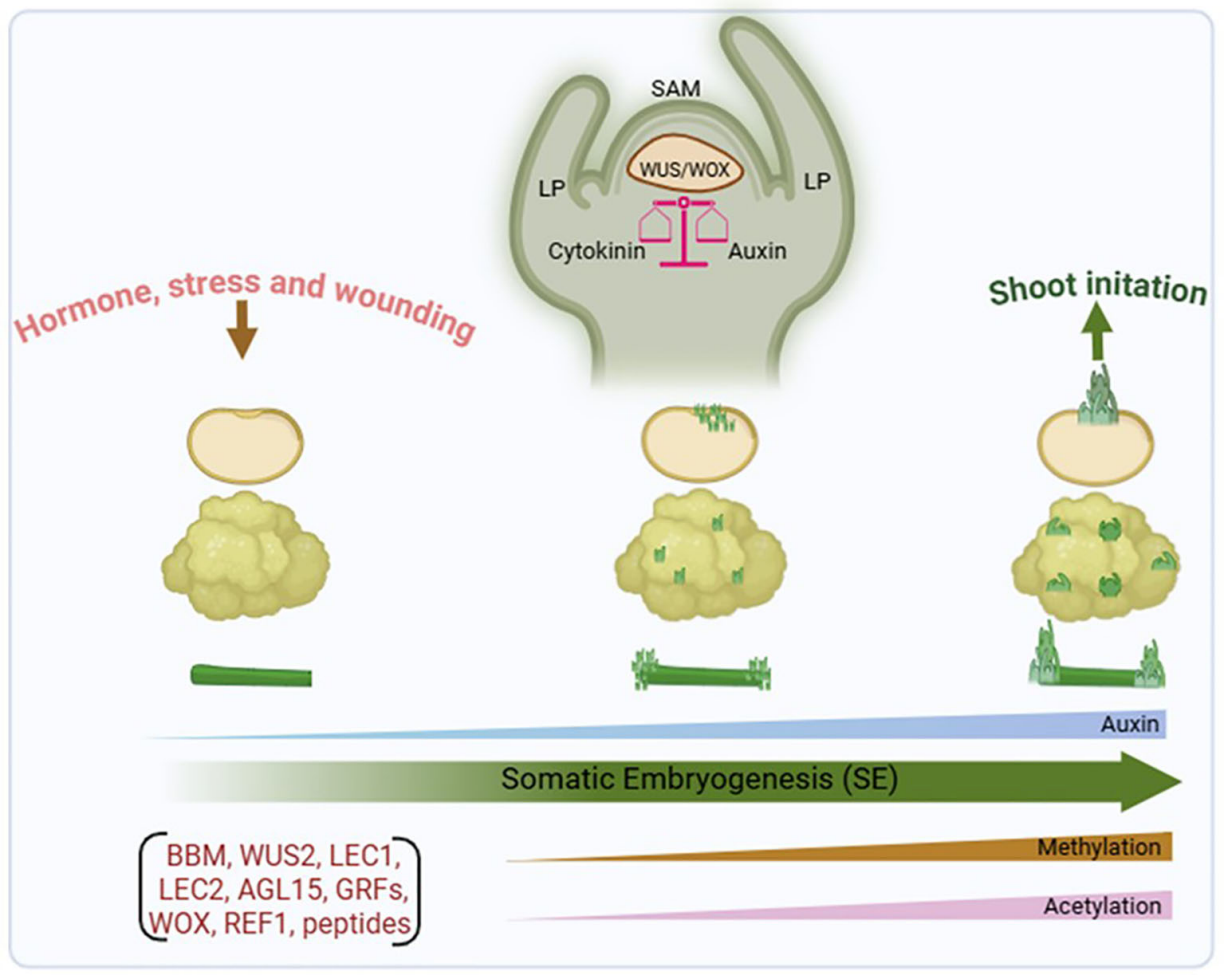
The diagram illustrates the process of somatic embryogenesis in plants. Number of factors involved for embryogenesis process, including transcription factors, epigenetic changes (DNA/histone methylation and histone acetylation), and hormones. The *WUS*/*WOX* is crucial for spanning hormonal balance in the shoot meristem region and assists in maintaining the shoot apical meristem (SAM) and embryonic development process. LP (Leaf primordia), BBM (BABY BOOM), WUS2 (WUSCHEL2), LEC (LEAFY COTYLEDON), AGL15 (AGAMOUS-Like 15), GRF1 (GROWTH-REGULATING FACTOR1), WOX (WUSCHEL-related homeobox) and REF1 (Regeneration Factor 1). Created in BioRender. (2026) https://BioRender.com/0vowex7.

Plant morphogenesis is a complex biological process that determines plant form and structure, encompassing growth, cell differentiation, and communication, and is influenced by biochemical signaling and genetic factors (Marconi and Wabnik, 2021). Foliage development is a great example of plant morphogenesis. The shoot apical meristem (SAM) produces leaves through shoot bud, which is controlled by phytohormones. Auxins and cytokinins have a major influence on the process that produces plant shoots and leaves. While cytokinin is essential for sustaining meristematic activity and cell proliferation, auxins stimulate organ initiation and patterning (Azizi et al., 2015; Kean-Galeno et al., 2024). Auxin plays a vital role in the development of the shoot apical meristem and its gradients, formed by transport and local biosynthesis, affect the spatial organization of the SAM, leading to the formation of new organs such as leaves at specified sites (Demesa-Arevalo et al., 2024). The development and function of the SAM are regulated by a complex interplay of hormones and developmental genes or regulators. Cytokinins are vital for maintaining the stem cell population in the shoot apical meristem (SAM) and promoting cell division. They work with auxin to regulate meristem activity and influence the expression of genes like *WUSCHEL* (*WUS*), essential for stem cell maintenance. Cytokinin signaling gradients, shaped by epidermal expression and transport, contribute to SAM spatial organization. The complex interaction between auxin and cytokinin affects cell fate, growth, and patterning, while gibberellins enhance cell division and elongation, contributing to plant height (Kean-Galeno et al., 2024; Demesa-Arevalo et al., 2024). Synthetic plant hormones can be used to precisely manipulate *in vitro* somatic embryogenesis processes, and somatic embryo development (Fehér, 2015). This model system serves as an excellent platform for investigating early plant development, pinpointing essential genes associated with embryogenesis, and identifying gene regulatory networks (Rose and Nolan, 2006; Horstman et al., 2017). During embryogenesis, somatic cells undergo multiple stages, including dedifferentiation, totipotency, differentiation, and embryo maturation, and these processes reprogramme gene expression both globally and locally in response to external cues and cellular morphogenic signals. In the past two decades, advancements in genomic technologies have facilitated the identification of essential genes that regulate somatic embryogenesis called morphoregulators (MRs) and morphogenic genes (*BBM*, *WUS2*, *LEC1*, *LEC2*, *AGL15*, *GRFs*, *WOX*, *REF1*, etc) (**Figure 2**). These MRs have been applied to recalcitrant genotypes, resulting in favorable outcomes for genotype-flexible transformation into a wide variety of crops (Gordon-Kamm et al., 2019; Maren et al., 2022; Chen et al., 2022a; Lee and Wang, 2023).

### 4.1 WUSCHEL

*WUSCHEL* (*WUS*) genes are homeodomain TFs with key roles in development and regulate the maintenance of the stem cell population in the SAM, as reported in *Arabidopsis* (Zuo et al., 2002; Jha et al., 2020). *WOX* are another class of WUS-related homeobox TFs that have similar developmental roles, including stem cell identity maintenance and embryo development (Van Der Graaff et al., 2009; Wang et al., 2022b; Fambrini et al., 2022). Due to their regulatory roles in axis determination and stem cell maintenance, *WUS*/*WOX* genes are particularly useful in plant transformation systems, specifically in promoting meristem and somatic embryo development. *WUS* overexpression enhances somatic embryogenesis in various dicot species, including Medicago (Tvorogova et al., 2019), tobacco (Rashid et al., 2007), white spruce (Klimaszewska et al., 2010) and coffee (Arroyo-Herrera et al., 2008). Expression of *ZmWUS2* promotes somatic embryogenesis in recalcitrant maize varieties (Lowe et al., 2016; Zuo et al., 2002). However, high expression levels of the *WUS2* gene resulted in a friable callus and pleiotropic effects during tissue culture plant development (McFarland et al., 2023; Gordon-Kamm et al., 2019). To address the issue, the *WUS2* gene was employed under the weak maize auxin-inducible (Axig1) promoter in conjunction with *BBM1* under strong expression, enabling effective transformation without detrimental effects on plant development (Lowe et al., 2018). The aforementioned issue was addressed through a non-integrating *ZmWUS2* based transformation system known as altruistic transformation. This method has been demonstrated in maize (Hoerster et al., 2020) and *Sorghum bicolor* (Che et al., 2022; Aregawi et al., 2022; Nelson-Vasilchik et al., 2022), utilizing the non-cell-autonomous movement of WUS protein via plasmodesmata to promote cell proliferation in adjacent cells (Fuchs and Lohmann, 2020). Additionally, the use of inducible site-specific recombinase CRE-loxP for the excision of morphogenetic genes (*WUS2* and/or *BBM*) after transformation was employed to produce fertile T0 plants to tackle the aforementioned problem (Wang et al., 2020b). Further developmentally regulated promoters (Ole, Glb1, End2, and Ltp2) were found to facilitate the excision of morphogenetic genes in early embryo development. The strategy resulted in 25–100% of excised events (Wang et al., 2020b).

*ZmWOX2a* is another morphoregulator that does not induce pleiotropic effects. Overexpression of this gene promotes the development of somatic embryos and the formation of embryogenic calli, making it an interesting candidate for the application of more versatile maize transformation methods using immature embryos (McFarland et al., 2023). Similarly, wheat *TaWOX5* surmounts genotype dependence and enhances transformation efficiency in wheat (60% across 21 varieties), rye, barley, and maize (Wang et al., 2020b). The Hairy Root-to-Shoot Conversion (HRC) method has been employed to generate shoots from hairy roots in cotton and citrus (Cui et al., 2020; Ramasamy et al., 2023). The combination of HRC and MRs approaches was used in apple, using the *MdWOX5* gene under an estradiol-inducible promoter to achieve a high frequency of shoot regeneration and develop transgenic plants from initial leaf explants. Furthermore, the combination of *ZmWUS2* and *IPT* resulted in a 29-40% increase of shoot induction frequency using HRC methods in radish (Yi et al., 2024).

In light of the importance of the *WUS* and *WOX* gene families, it is crucial to identify more suitable gene family members for use in enhancing plant regeneration and transformation. A phylogenetic analysis of *WOX*/*WUS* was conducted using the maximum likelihood method, which predicted the orthologs of these genes. These orthologs can be utilized individually or in combination, making them most suitable for transformation systems aimed at enhancing plant regeneration potential in recalcitrant crops (Youngstrom et al., 2025). Studies revealed that WUS plays a crucial role in the regulation of shoot apical meristem (SAM) maintenance, *WOX2* is essential for embryonic development, and WOX5/7 regulates the maintenance of root stem cell populations (Haecker et al., 2004; Sarkar et al., 2007; Zhang et al., 2017a). Research indicates that *AtWOX8* or *AtWOX9* members can rescue *wus* null mutants (Dolzblasz et al., 2016). Additionally, *WOX8*/*9* has been shown to induce somatic embryogenesis in tobacco and Medicago plants (Kyo et al., 2018; Tvorogova et al., 2019; Yakovleva et al., 2024). These investigations indicate that a controlled and limited expression of *WUS* orthologs can be an effective strategy for successfully inducing somatic embryogenesis while avoiding pleiotropic effects. *WOX2* and *WOX5* orthologues, on the other hand, can easily be used in the transformation of several monocot and dicot plants in a straightforward manner. Consequently, *WUS* orthologs may be more effectively employed through well-regulated expression methods, such as inducible promoters or excision post-somatic embryogenesis, instead of depending on robust constitutive promoters. In contrast, employing *WOX2* or *WOX5* members would be more straightforward, since they do not require the removal of MRs for normal plant development.

### 4.2 BABY BOOM

*BABY BOOM* (*BBM*) gene, a member of the *AP2 (APETALA2)/ERF (ETHYLENE RESPONSIVE ELEMENT BINDING FACTOR*) superfamily of transcription factors, has been gained attention in plant transformation research from its first time discovered in *Brassica napus* which is regulator of plant cell totipotency and produce somatic embryos in turn produce plants (Boutilier et al., 2002). This process involves somatic embryogenesis and organogenesis through the auxin signaling pathway. It has been demonstrated that ectopic expression of *BBM* induces somatic embryogenesis without the need for exogenous plant growth regulators or stress conditions (Boutilier et al., 2002; Horstman et al., 2017), but hormone addition improves somatic embryogenesis process (Boutilier et al., 2002; Lee and Wang, 2023). Research indicates that elevated expression of *BBM*, which is involved in embryo development, can occasionally result in pleiotropic effects that interfere with normal plant development (Lee and Wang, 2023; Maren et al., 2022; Chen et al., 2022a). *BBM* orthologues have been utilized in various crop systems to enhance somatic embryogenesis and transformation efficiency in multiple species, including *Rosa canina* (Yang et al., 2014a), *Theobroma cacao* (Florez et al., 2015), *Capsicum annuum* (Heidmann et al., 2011), *Coffea arabica* (Silva et al., 2015), *Sorghum bicolor* (Lowe et al., 2016; Mookkan et al., 2017), *Saccharum officinarum* (Lowe et al., 2016), *Oryza sativa* (Lowe et al., 2016) and *Musa acuminata* (Shivani et al., 2017). Considering the fact that *WUS* and *BBM* individually promote varying degrees of somatic embryogenesis, it has been recommended to combine both MRs in order to increase the rate of genotype-independent transformation of maize using *BBM* and *WUS2* genes (Lowe et al., 2016). The Lowe et al. group has developed a strategy that expresses *BBM* under the maize phospholipid transferase protein promoter (PLTP) and *WUS2* under the maize auxin-inducible gene 1 promoter (Axig1), which promotes high transformation efficiency in several elite maize varieties (Lowe et al., 2018). Monocot transformation primarily utilizes immature embryo explants. Generally, monocot transformation uses immature embryo explants. Having year-round access to immature embryo explants makes the transformation process difficult. Since leaf bases maintain embryonic cell mass, Wang et al. (2023) developed a leaf-based transformation system using the combination of *ZmBBM* and *ZmWUS2* expression system and established the system and successfully generated plants in different crops species including teff, maize, sorghum, foxtail millet, pearl millet, rye, barley, rice. Additionally, the *ZmBBM1* and *ZmWUS2* expression systems have been used to develop highly efficient transformants in hexaploid wheat (Johnson et al., 2023). Based on the results of these studies, it can be concluded that the combination of growth regulators is capable of promoting high efficiency transformation and further overcoming the barriers to genetic transformation in numerous elite crops, which have previously been difficult to transform.

### 4.3 GRF-GIF

Plant growth-regulating factors (GRFs) play an important role in regulating plant growth, including the development of leaves (Horiguchi et al., 2005; Kim and Lee, 2006; Omidbakhshfard et al., 2015). GRFs physically interact with GRF-interacting factors (GIFs), a group of transcriptional coactivators, forming a complex that recruits chromatin remodeling complexes (SWI2/SNF2) to facilitate chromatin opening and enhance transcription, thereby promoting cell division and leaf development (Vercruyssen et al., 2014; Debernardi et al., 2014, Debernardi et al., 2020). The expression of various *GRFs* in plants is regulated by the microRNA miR396. *GRFs* exhibit higher expression levels in actively growing tissues, such as roots and shoots, particularly in regions of cell proliferation (Kim et al., 2003; Rodriguez et al., 2010). In land plants, there are typically 8–20 *GRF* members present in their genomes, which is highly conserved (Omidbakhshfard et al., 2015). Research indicates that *GRF5* facilitates organogenesis in various dicotyledonous plant species (Kong et al., 2020). A strong expression of the *GRF5*-mediated transformation and regeneration system has been developed in multiple dicot and monocot plant species, showing improved regeneration efficiencies in soybean, canola, sunflower, sugar beet, and maize (Kong et al., 2020). *GRFs* constitute a multi-gene family within each crop genome and exhibit diverse functions. *Arabidopsis GRF5* overexpression significantly improved sugar beet transformation, however sugar beet *GRF5* showed no significant improvement. Furthermore, the overexpression of *AtGRF5* did not exhibit pleiotropic effects in canola and sugar beets; however, it was occasionally linked to abnormal root development in soybeans (Kong et al., 2020). Studies indicates that the overexpression of *GRF5*–*GIF1* fusion product boosts both organogenesis and embryogenesis, demonstrating greater efficiency in plant regeneration and transformation compared to the overexpression of *AtGRF5* alone (Debernardi et al., 2020; Kong et al., 2020).

Study conducted by Debernardi and coworkers, developed a wheat genome editing construct using a miR396 resistant variant of wheat *GRF4* in combination with *GIF1* under strong expression of maize ubiquitin promoters. The TaGRF4-TaGIF1 chimera enhanced regeneration and transformation efficiency by approximately 7.8-fold when utilizing wheat immature embryos as explants (Debernardi et al., 2020). Additionally, the study showed that chimeric *TaGRF4*-*TaGIF1* constructs improved regeneration efficiency in *Triticum durum*, *Triticum aestivum*, triticale, and rice. Interestingly, Debernardi et al. (2020) found that TaGRF4-TaGIF1 chimeras enhanced shoot regeneration without including cytokinin in their media for shoot induction. In contrast to *BBM* or *WUS*-mediated transformation, the overexpression of *GRF* orthologs does not exhibit pleiotropic effects and results in the generation of fertile transformed plants (Debernardi et al., 2020; Kong et al., 2020; Qiu et al., 2022). In recent studies, the *Agrobacterium* ternary vector system was combined with GRF-GIF chimeras, which resulted in an additive effect in the transformation of sorghum and maize (Li et al., 2024; Vandeputte et al., 2024). The *TaGRF4*-*OsGIF1* chimera construct in sorghum, regulated by the *Arabidopsis* UBQ10 promoter, was combined with the pVS1-VIR2 ternary helper plasmid (Zhang et al., 2019b), resulting in a 7.71-fold increase in transformation efficiency (Li et al., 2024). It has been shown that using the pVS1-VIR2 ternary helper in maize B104 transformations with immature embryos increases the transformation frequency from 2.3 to 8.1% (3.5-fold), and adding *ZmGRF1*-*ZmGIF1* chimeras increases the transformation frequency by another 3.5-fold from 13.6 to 47.4% (Vandeputte et al., 2024). The GRF4-GIF1 fusion demonstrated functionality in dicots as well. GRF4-GIF1 chimeras showed about a five-fold increase in regeneration rate in citrus or grape (Debernardi et al., 2020). Besides the GRF4-GIF1 chimera, other GRF-GIF fusion proteins, such as GmGRF3-GmGIF1, have been effectively utilized to improve transformation in soybean (Zhao et al., 2025), grape (*VvGRF4*-*VvGIF1*), and tomato (*SlGRF8*-*SlGIF4*) and *SlGRF12*-*SlGIF4* chimeras in lettuce (Bull et al., 2023). The studies indicate that crop-specific combinations of GRF-GIF represent a promising approach to enhance regeneration efficiency in various monocot and dicot species.

### 4.4 Other class of morphoregulators

A family of *LEAFY COTYLEDON*s genes called *LEC1*, *LEC2*, and *FUS3* (*FUSCA3*) function as transcription factors crucial for embryogenesis and maturation processes (Chen and Du, 2022). LEC transcription factors are involved in embryonic development, seed maturation, and stalk growth in Arabidopsis thaliana. LECs and several other transcription factors establish a complex network that regulates numerous facets of plant growth and development. *LEAFY COTYLEDONs* (*LEC1*, *LEC2*, and *FUSCA3*) are the key genes in plant embryonic development (Chen and Du, 2022; Guo et al., 2022). During embryonic morphogenesis, the *LEAFY COTYLEDONs* determine the fate of stalk cells and control cotyledon characteristics (Chen and Du, 2022). *LEAFY COTYLEDONs* (*LEC1*, *LEC2*, and *FUSCA3*) are essential genes in plant embryonic development that interact to activate target genes and facilitate the development process in plants (Santos-Mendoza et al., 2008; Chen and Du, 2022). *LEC1* promotes embryonic development and activates maturation and storage genes, as well as lipid accumulation in vegetative organs when expressed ectopically (Mu et al., 2008; Manan et al., 2023; Zhang et al., 2024a). Previous study indicates that 35S:PaLEC1-GFP transgenic lines induce various types of somatic embryonic structures and tissue clusters containing multiple shoot apical meristems (Chen et al., 2019). Citrus sinensis *LEC1* overexpression in sweet orange resulted in the formation of embryo-like structures in the typically recalcitrant epicotyls after one month, and subsequently, after an additional two months on elongation medium, shoots with aberrant leaves were produced (Zhu et al., 2014). Moreover, the overexpression of *PaHAP3A*, a member of the *LEC1*/*L1L* family, did not lead to the formation of ectopic somatic embryos in vegetative tissues (Uddenberg et al., 2016). Transgenic lines introducing 35S::*AtLEC2* into *lec2–1* and *lec2–5* mutant lines of Arabidopsis, leading to the formation of ectopic somatic embryos capable of germination and plant production (Stone et al., 2001). LEC2 appears to promote more complete somatic embryo formation than *LEC1*, *L1L*, or *FUS3*. This aligns with evidence suggesting that *LEC2* operates upstream of and activates both LEC1 and FUS3. However, the resultant plant phenotypes were abnormal, and the authors did not address fertility (Santos Mendoza et al., 2005). Consequently, the existing fragmented research on the role of *LECs* and their molecular mechanisms in linking embryonic and vegetative growth periods with the reproductive stage requires increased attention in future studies.

SOMATIC EMBRYOGENESIS RECEPTOR KINASE (SERKs) belongs to the leucine-rich repeat receptor-like kinase (LRR-RLK) family, play vital roles in morphological development, stress and defense responses, and signal transduction (Arimarsetiowati et al., 2024). The *SERK* gene is pivotal in embryo formation from single somatic cells. *SERK* genes were first linked to somatic embryogenesis, with later studies showing their role in somatic embryogenesis and organogenesis across various species including sunflower (Thomas et al., 2004), Ocotea (Santa-Catarina et al., 2004), citrus (Shimada et al., 2005), grapevine (Schellenbaum et al., 2008), potato (Sharma et al., 2008), wheat (Singla et al., 2008) banana (Huang et al., 2010). In vanilla, enhanced *SERK* expression is linked to embryogenesis (Ramírez-Mosqueda et al., 2018). *SERK1* overexpression of the plant gene results in enhanced formation of somatic in Arabidopsis without changes in plant phenotype (Hecht et al., 2001). The overexpression of the *Coffea canephora SERK1* gene during *in-vitro* somatic embryogenesis significantly boosts the production of somatic embryos, increasing it by twofold (Pérez-Pascual et al., 2018). AGL15, a component of the SERK1 protein complex, demonstrated a response similar to that of SERK1 (Karlova et al., 2006). Overexpression of the *AGL15* gene enhanced secondary somatic embryo formation in Arabidopsis (Harding et al., 2003), boosted somatic embryo production in soybean (Thakare et al., 2008), and further improved embryogenic callus formation in cotton (Yang et al., 2014b). Additional investigation into the biological roles of the *SERK* gene in crops, along with its validation, would improve the development of effective regeneration systems, thereby enhancing understanding and application of transformation techniques.

MRs have the ability to induce the development of axillary meristems along with the development of shoots and roots. Several basic helix-loop-helix transcription factors are involved in the regulation of axillary meristems. It has been shown that the *LAX PALICLE1* of wheat (*TaLAX1*) and rice (*OsLAX1*) plays a significant role in the formation of axillary meristems and grain production (Gallavotti et al., 2004; Oikawa and Kyozuka, 2009). There was a considerable increase in regeneration frequency when the orthologs of *TaLAX1* were overexpressed in both maize and soybean, suggesting that *TaLAX1* and its orthologs could be manipulated to improve transformation in both monocots and dicots (Yang et al., 2024). By overexpressing *TaLAX1a* in wheat varieties, it was found that florets per spikelet increased, regeneration improved, and gene editing frequencies increased as well (Yu et al., 2024). Based on the results of these studies, it appears that overexpression of *TaLAX1* results in enhanced gene expression of *TaGRFs* and *TaGIF1*, which ultimately improve regeneration and transformation in wheat, as observed by Debernardi et al. (2020).

*KNOTTED1* (*KN1*) is a homeodomain transcription factor that plays a crucial role in the maintenance of shoot stem cells in plants (Kitagawa et al., 2022). Tobacco plants that overexpression *ZmKN1* constitutively can dramatically enhance shoot organogenesis compared to control ones (Luo et al., 2006). Tobacco plants overexpressing *NTH15* and *NTH20* exhibited pleiotropic phenotypes but also formed ectopic shoot meristems on the leaves to some extent. Nonetheless, the other members exhibited significant abnormalities in tobacco (Nishimura et al., 2000). A *KNOTTED1-like homeobox* (*KNOX*) gene, *OSH1*, is induced by cytokinin, leading to the regeneration of shoots from calli mass in rice (Naruse et al., 2018).

*AGAMOUS-LIKE15* (*AGL15*), which is a MADS-domain protein, is a crucial player both in *Arabidopsis* and soybean in the development of the somatic embryo (Zheng et al., 2013). It has been demonstrated that ectopic constitutive expression of AGL15 promotes somatic embryogenesis in *Arabidopsis*, soybean, and cotton (Harding et al., 2003; Thakare et al., 2008). In contrast, mutants of *AGL15* and its close relative *AGL18* significantly impede somatic embryogenesis in *Arabidopsis* (Zheng and Perry, 2014). In soybeans and *Arabidopsis*, *AGL15* and *AGL18* also promote somatic embryonic development when expressed ectopically (Zheng and Perry, 2014; Paul et al., 2021).

### 4.5 Utilization of small peptides for plant regeneration

Plants use diverse regeneration strategies after injury, primarily by regenerating their shoot or root apical meristem following localized damage. This enables the formation of new organs or entire plants from small tissue fragments or individual cells through *de novo* organogenesis or somatic embryogenesis. *De novo* organogenesis reactivates cellular proliferation at wound sites that aids regeneration (Yang et al., 2024). *WOUND-INDUCED DEDIFFERENTIATION* (*WIND*) AP2/ERF transcription factors, including *WIND1* and its homologs *WIND2*, *WIND3*, and *WIND4*, are key regulators of wound-induced cellular reprogramming. These factors promote dynamic transcriptional changes and regulate multiple physiological responses following wounding. *WIND1* facilitates callus formation and shoot organogenesis through the direct activation of a shoot meristem regulator (Iwase et al., 2011, Iwase et al., 2017). Ectopic expression of *AtWIND1* in *B. napus* increased shoot regeneration rate by 24- to 47-fold from segments of hypocotyl (Iwase et al., 2015). *WIND1* is involved in plant regeneration and functions downstream of the REF1-PORK1 module. The transgenic overexpression of *ZmWIND1* led to a 3–4 fold increase in callus induction in maize (Jiang et al., 2024a). Additionally, *ESR1* functions downstream of *WIND1 and ESR1* does not provide feedback to cytokinin-responsive genes (Iwase et al., 2011). Ectopic expression of *ESR1* or its paralog *ESR2* alone can induce shoot organogenesis (Ikeuchi et al., 2019) In Arabidopsis, *ESR1* expression increased shoot regeneration efficiency by 18.2% via the activation of *CUC1*, *WUS*, and *STM* (Iwase et al., 2021). Researchers found that stable transgenic tomatoes and peppers expressing *PLT1*/*WOX5*/*WIND1* can boost regeneration efficiency without external hormones (Wittmer et al., 2025). Further research on WIND could help identify genes directly targeted by *WIND1*, shedding light on how *WIND1*-mediated signaling controls the transcription of key regulators in regeneration and uncovering a critical transcriptional mechanism linking the wound response to organ regeneration in plants.

Systemin and its precursor gene *PROSYSTEMIN* (*PRS*) mainly regulate systemic defense responses. CRISPR-Cas9-mediated knockout mutants of *PRS* and the systemin lacked systemic defense responses (Yang et al., 2024). Yang and coworkers show that reducing the tomato *Pep* (*SIPep*) precursor gene or its receptor gene eliminates regeneration capacity in wound-induced callus formation and shoot regeneration. In contrast, overexpression enhances regeneration capacity. Exogenous application of the SlPep peptide also significantly boosts regeneration capacity. Subsequently, SlPep was named REGENERATION FACTOR1 (REF1) a plant-derived polypeptide that functions as a localized wound signal molecule, playing a pivotal role in tissue regeneration. The REF1 peptide has been demonstrated to boost regeneration and transformation efficiencies in maize, soybean and wheat, suggesting its potential to significantly improve the recalcitrant crop regeneration and transformation processes (Yang et al., 2024). *REF1* signaling activates *SlWIND1*, additionally, *SlWIND1* amplifies the *REF1* signal by promoting the transcription of the *REF1* precursor gene. Thus, REF1 functions as a local wound signal that supports plant regeneration. *REF1* activates downstream *MAPK* cascades through strong binding to its receptor ORTHOLOG RECEPTOR-LIKE KINASE 1 (PORK1), resulting in the expression of *SlWIND1*. *SlWIND1* facilitates callus development and organ regeneration through its interaction with wound-responsive elements in the proline-rich protein (PRP) promoter, thereby establishing a “REF1-PORK1-WIND1-PRP” positive feedback loop. This loop enhances regeneration signals and preserves equilibrium in cells (Yang et al., 2024). A dose-dependent effect was noted with the exogenous application of the REF1 peptide, which enhanced callus formation and shoot regeneration in *prp* and *pork1* mutants, while also addressing regeneration defects in these mutants (Yang et al., 2024; Schaller, 2024). Recent studies indicate that exogenous supplementation of 10 nmol CaREF1 (10 nmol/L) enhanced regeneration by 2.1-fold and increased shooting frequency by 4.4-fold, resulting in 100% editing efficiency in CRISPR/Cas9 integrated pepper plants (Wang et al., 2025). The new generation approach in REF1 application offers a robust platform for molecular breeding, especially in recalcitrant crops like soybean, wheat, and maize and enhances genetic transformation efficiency by 4–5 times, addressing the transformation bottleneck in these economically important species for food security (Trösch, 2024). The identification of REF1 offers a viable approach to augment the regeneration potential of recalcitrant crops, consequently enhancing their transformation efficiency.

Small signaling peptides are vital for plant growth, development, and environmental responsiveness, with the CLAVATA3/EMBRYO SURROUNDING REGION-RELATED (CLE) family being one of the most studied (Kang et al., 2022b). *De novo* shoot regeneration involves forming new shoots from explants lacking pre-existing shoot meristems, requiring coordination among signaling molecules and transcription factors like CLE peptides and WUSCHEL (WUS). CLE peptides regulate stem cell homeostasis, vascular differentiation, and root cap development, and are promising targets for improving genetic transformation and genome editing efficiency (Kang et al., 2022b; Niu et al., 2025). *CLE* genes encode precursor proteins with an N-terminal signal sequence for secretion, a variable central region, and a conserved C-terminal CLE domain, often modified post-translationally to form functional polypeptides (Ji et al., 2025). These genes are crucial for stem cell regulation (Kang et al., 2022b). The expression of *CLE* varies during the phases of shoot regeneration (Wang et al., 2022c).The transcription of *CLE1*-*CLE7* genes is markedly increased by callus- or shoot-inducing medium (Kang et al., 2022b). CLE1-CLE7 (Kang et al., 2022b) and CLE9/10 (Glazunova et al., 2025) peptides negatively impact shoot regeneration. While *cle1–7* septuple mutant exhibits enhanced regeneration capacity and an increased number of adventitious shoots formation (Kang et al., 2022b). Mutations in the single cle locus have minimal impact on *de novo* shoot regeneration, while both cle4 and cle7 mutants demonstrate enhanced *de novo* shoot formation (Kang et al., 2022b). The overexpression of *CLE4*/*CLE7* significantly inhibits regeneration (Kang et al., 2022b). Exogenous administration of synthetic CLE peptides (CLE1–7) exhibits a dose-dependent reduction of *de novo* shoot regeneration. Recent research suggests that CLE9/10 peptides suppress *de novo* shoot regeneration but do not influence callus formation (Glazunova et al., 2025). The overexpression of *CLE41*/*42* promotes axillary bud formation, while the external application of either tracheary element differentiation inhibitory factor (TDIF) or CLE42 peptide to the wild type similarly induces an increase in bud emergence (Yu et al., 2025). CLE peptides facilitate signaling via CLV1 and BAM1 receptors, establishing a CLE-WUS regulatory axis that inhibits WUS expression and restricts adventitious shoot formation (Zhang et al., 2024b). Inhibition of CLE peptides alleviates this suppression, thereby improving regeneration and the efficiency of genetic transformation. CLE signaling modules, typically consisting of CLE peptides, receptor-like kinases (RLKs), and WUSCHEL-related HOMEOBOX (WOX) transcription factors, are key regulators of stem cell homeostasis in various plant meristems, including shoot apical, root apical, axillary, vascular, and nodule meristems (Olsson et al., 2019; Fletcher, 2020). Mutants of *clv1* and *bam1* exhibit enhanced shoot regeneration abilities and demonstrate insensitivity to the inhibitory effects of CLE1-CLE7 peptides on shoot regeneration in arabidopsis. This confirms the CLE1-CLE7 signal negatively regulates *de novo* shoot regeneration via CLAVATA1 (CLV1) and BARELY ANY MERISTEM1 (BAM1) receptors (Glazunova et al., 2025). Furthermore, the CLE-CLV1/BAM1 module suppresses the expression of *WUSCHEL*, a critical regulator regeneration, thereby regulating the possibility for shoot formation (Shpak and Uzair, 2025).

### 4.6 Methods to discover novel regeneration morphoregulators

RNA-seq, ATAC-seq, and CUT&Tag are effective methodologies employed to examine the interactions among multiple levels of cellular information within cells. RNA-seq offers insights into gene expression, while ATAC-seq identifies accessible regions of chromatin, suggesting possible regulatory elements. CUT&Tag technology demonstrates chromatin accessibility while targeting specific proteins or histone modifications (Ouyang et al., 2021). Incorporating these methods helps researchers understand gene regulation and cellular function. In wheat, RNA-seq, ATAC-seq, and CUT&Tag have been used to identify candidate MRs to understand transcriptional regulatory networks that regulate embryonic callus formation and organogenesis (Liu et al., 2023b). This study revealed 446 core TFs involved in regeneration, as well as transcriptomic and epigenetic changes during callus maturation and shoot regeneration. As a result of the comparison of these 446 core TFs between *Arabidopsis* and wheat transcriptomes, TaDOF3.4 and TaDOF5.6 were identified as candidate MRs for wheat varieties with high and low regeneration genotypes (Liu et al., 2023b). In subsequent experiments, overexpression of the *TaDOF5.6* or *TaDOF3.4* genes under the maize Ubi promoter significantly improved wheat transformation efficiency (Fielder and Jimai22), which displayed a normal plant phenotype and seed production rate, indicating their potential use to boost wheat transformation (Liu et al., 2023b). Further, according to a recent study, 53 plant MRs have homologs in a wide variety of species, and their expression increases during embryonic development, embryogenic callus formation, and somatic embryogenesis (Jiang et al., 2024a), including *ZmBBM*, *TaWOX5a*, *TaDOF5.6*, which have been previously shown to improve transformation efficiency (Liu et al., 2023b; Lowe et al., 2016, Lowe et al., 2018; Wang et al., 2023, Wang et al., 2022b). Based on the results of this study, a novel morphoregulator of the ERF/AP2 TF family member is identified as maize *WIND-INDUCED DIFFERENTIATION 1* (*ZmWIND1*) (Jiang et al., 2024a). It has been reported that the *Arabidopsis* ortholog of *ZmWIND1* (*AtWIND1*) promotes callus formation and organogenesis through direct activation of *ENHANCER OF SHOOT REGENERATION1* (*ESR1*) (Iwase et al., 2017). *ZmWIND1* is negatively regulated by *ZmSAUR15*, which inhibits embryonic callus induction in maize. It was found that the loss of *ZmSAUR15* function was associated with an increase in *ZmWIND1* expression, which was associated with an increase in embryonic callus formation (Wang et al., 2022d). When *ZmWIND1* was strongly overexpressed, regeneration and transformation frequencies were increased by approximately two to three-fold in two maize varieties (Jiang et al., 2024a).

## 5 Strategies for using morphogenic regulators in transformation and gene editing

**Figure 3:**
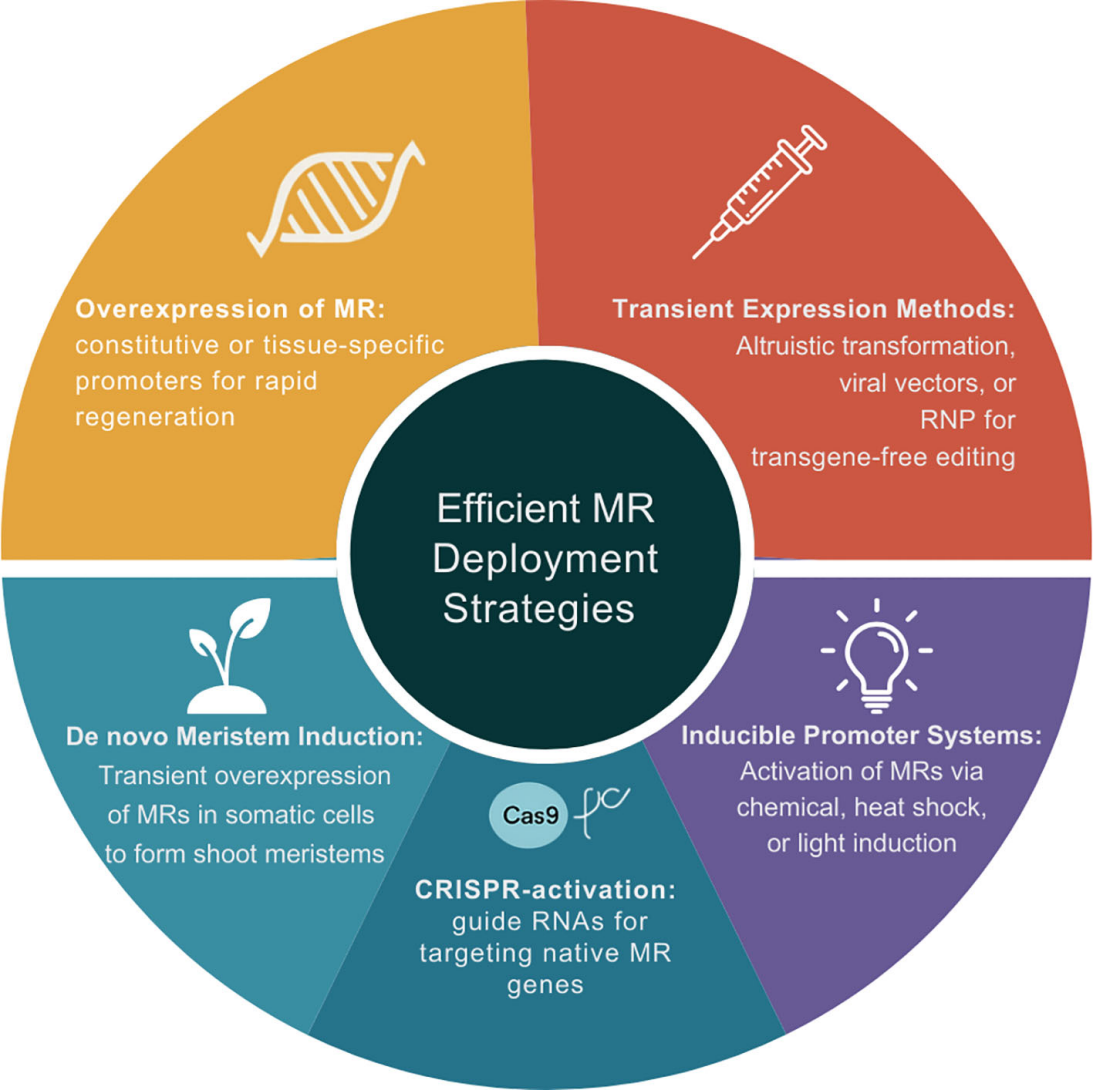
Deployment strategies for morphoregulatory (MR) genes to enhance plant transformation and genome editing. The illustration summarizes five approaches used to maximize regeneration efficiency while minimizing pleiotropic effects associated with morphogenic gene expression. These include: (i) constitutive or tissue-specific promoters for spatial control of MR expression; (ii) transient expression methods such as altruistic transformation or direct delivery; (iii) inducible promoter systems triggered by chemical, heat, or light stimuli; (iv) RNA-based delivery systems utilizing CRISPR activation of endogenous MR genes; and (v) *de novo* meristem induction through transient MR expression in somatic cells. Image generated on Canva (https://www.canva.com/design/DAGqZjdHQMY/8sZdpSBeaoTT-frOpwBhLQ/edit).

While the identification and characterization of developmental regulators that could efficiently enhance the transformation efficiency of recalcitrant species was a significant breakthrough, the greatest bottleneck was their efficient deployment in a way that harness the benefits but reduces the associated tradeoffs. For instance, numerous studies reported that the constitutive overexpression of MRs resulted in detrimental effects and developmental abnormalities (Gordon-Kamm et al., 2019). As such, researchers have come up with the strategies outlined below for delivering, expressing and regulating MRs, including constitutive vs. tissue-specific promoters, transient expression methods, inducible systems, and RNA-based delivery (Aesaert et al., 2022; Wang et al., 2020a) (**Figure 3**).

### 5.1 Overexpression of MRs under constitutive promoters and spatial/temporal control via tissue-specific promoters and excision

The most robust, simplest and widely applicable strategy involves inclusion of the MRs expression cassette in the T-DNA of plant transformation vectors and expressing them under strong constitute promoters such as the maize Ubiquitin1 (ZmUbi) or the cauliflower mosaic virus (CaMV) 35S promoter. This strategy offers the advantage of simplicity of delivery and immediate robust expression since every plant tissue expresses constitutive promoters (Gordon-Kamm et al., 2019). For instance, Lowe et al. (2016) demonstrated enhanced recovery of transgenic plants, particularly in recalcitrant or marginally transformable maize, rice, sorghum, and sugarcane varieties, following constitutive expression of *WUS* and *BBM* under the maize ubiquitin promoter. Even more interesting is that the procedure enabled direct *Agrobacterium*-mediated transformation of mature seed-derived embryo axis or leaf segments, without an intervening callus or meristem culture step. However, most of the regenerated plants had undesired pleiotropic effects ranging from altered lateral growth to sterility, since the MRs remained active beyond the regeneration stage (Jiang et al., 2024b) (**Table 1**).

**Table 1:** Comprehensive overview of constitutive expression strategies using morphogenic regulators across diverse crops to enhance somatic embryogenesis, organogenesis, and transformation efficiency.

Strategy	Morphogenic regulators	Crop(s)	Key advantages	Limitations/considerations	Promoter(s)	References
Constitutive expression	*BnBBM1*, *BnBBM2*, *AtBBM*	*Brassica*, *Arabidopsis*	Induced spontaneous somatic embryo formation, ectopic shoot development, enhanced cell proliferation, and hormone-free regeneration of explants.	Pleiotropic effects: neoplastic growth, dwarfism, altered leaf/flower morphology, delayed flowering, increased anthocyanins, wax deficiency, poor root growth, compromised vegetative development, and reduced male/female fertility.	Double-enhanced 35S + Alfalfa mosaic virus (AMV) translational enhancer; Sunflower UbB1 polyubiquitin	(Boutilier et al., 2002)
	*VvGRF4-GIF1*	*Fragaria vesca* (Hawaii 4)	Significantly improves regeneration efficiency	miRNA-resistant VvGRF4-GIF1 and TaGRF4-GIF1 variants caused abnormalities due to deregulated expression	CaMV 35S	(Sanchez et al., 2024)
	*AtBBM, PLT2*	*Arabidopsis*	Dose-dependent developmental outcomes: high expression promotes somatic embryogenesis, while low expression induces organogenesis; also activates LAFL gene expression.	Not specified	CaMV 35S	(Horstman et al., 2017)
	*BnBBM*	*Capsicum annuum* (sweet pepper)	Promotes somatic embryogenesis and enhances regeneration efficiency	Not specified	CaMV 35S	(Heidmann et al., 2011)
	*GmBBM1*	*Glycine max*, *Arabidopsis thaliana*	Induces somatic embryo formation and promotes embryo development	Delayed flowering, reduced seed set, enlarged floral organs, elongated cotyledons, post-germination somatic embryos, thick/short hypocotyls, and abnormal roots	Double CaMV 35S promoter with AMV translational enhancer	(El Ouakfaoui et al., 2010)
	*LdLEC1*	*Larix decidua*, *Arabidopsis*	Involved in somatic embryogenesis	Not specified	CaMV 35S	(Rupps et al., 2016)
	*RcBBM1*and *RcBBM2*	*Rosa canina*, *Arabidopsis*	Spontaneous somatic embryo formation and enhanced shoot regeneration	Pleiotropic effects: short roots, altered leaf morphology, delayed flowering	Cauliflower mosaic virus 35S	(Yang et al., 2014a)
	*TcBBM*	*Theobroma cacao*	Promotes hormone-free transition to embryonic state	Abnormal cotyledon development, serial initiation of multiple somatic embryos (meta-embryos)	CaMV 35S	(Florez et al., 2015)
	*AtBBM* and *BnBBM1*	Tobacco (*Nicotiana tabacum*)	Induces organogenesis; somatic embryogenesis upon cytokinin addition	Severe pleiotropic effects including dwarfism, loss of apical dominance, callus formation, leaf rumpling, floral abnormalities, and sterility	35S	(Srinivasan et al., 2006)
	*AtWUS*	*Arabidopsis thaliana*	Promote vegetative-to-embryonic transition and somatic embryo formation	Not specified	CaMV 35S	(Zuo et al., 2002)
	*AtWUS*	*Coffea canephora*	Strong enhancement of somatic embryo production (400x) and callus formation	Requires specific developmental stage and hormonal conditions for full embryogenic effect	CaMV 35S	(Arroyo-Herrera et al., 2008)
	*AtWUS*	Capsicum chinense	Induced formation of globular structures suggesting embryogenesis in a highly recalcitrant species	No complete regeneration or transformation system established; early-stage embryogenesis only	CaMV 35S	(Solís-Ramos et al., 2009)
	*AtWUS* and *AtSTM*	*Arabidopsis thaliana*	WUS and STM synergistically trigger organogenesis from non-meristematic tissue	Did not restore full meristem self-maintenance or correct phyllotaxy; Arabidopsis-specific	CaMV 35S	(Gallois et al., 2002)
	*GhWUS1a* and*GhWUS1b*	*Arabidopsis thaliana, Gossypium hirsutum*	Promotes embryogenesis and organogenesis without exogenous hormones; induces somatic embryos from non-embryogenic tissues	Overexpression of a repressor form (GhWUS-SRDX) blocked embryogenic callus formation in cotton, highlighting dosage and regulation sensitivity	CaMV 35S	(Xiao et al., 2018)
	*AtWUS*	*Gossypium hirsutum*	3× embryogenic explants; induced organogenesis in embryo-like tissues	No plant regeneration in recalcitrant variety under standard protocols; requires optimization of hormones	CaMV 35S	(Bouchabké-Coussa et al., 2013)
	*AtWUS*	*Gossypium hirsutum* (CRI12)	47.75% increase in embryogenic callus formation in a recalcitrant cultivar	Potential pleiotropic effects; evaluated only in callus stage	CaMV 35S	(Zheng et al., 2014)
	*GRF4–GIF1*	Wheat	7.8× transformation frequency in elite cv. Fielder, Marker-free selection, Applicable to recalcitrant genotypes, Shoots visible in 7–10 days vs. 3–4 weeks control	Yield-related tradeoff: exhibited a 23.9 % reduction in number of grains per spike	Maize Ubiquitin	(Debernardi et al., 2020)
	*GRF4–GIF1*	Triticale	Transformation in previously non-transformable genotypes, Faster shoot emergence	Limited data across cultivars	Maize Ubiquitin	(Debernardi et al., 2020)
	*GRF4–GIF1*	Rice	Faster regeneration, Shoots in 6–10 days vs. 15–20 days control, Increased number of transgenic plants	Not validated in japonica/indica breadth	Maize Ubiquitin	(Debernardi et al., 2020)
	*GRF-GIF* (Citrus)	Citrus	Improved shoot regeneration from epicotyls, Shoots in 12–14 days vs. 3–4 weeks control, First report of chimeric GRF-GIF effect in dicots	Chimera version not identical to wheat construct	CaMV 35S	(Debernardi et al., 2020)
	*AtGRF5 / BvGRF5-L*	Sugar beet	Enabled transformation of recalcitrant varieties; increased transformation efficiency	None reported	2×35S	(Kong et al., 2020)
	*AtGRF5/HaGRF5-L*	Sunflower	Improved transgenic shoot formation	None reported	2×35S	(Kong et al., 2020)
	*GmGRF5-L*	Soybean	Improved transgenic shoot formation	None reported	PcUbi4-2	(Kong et al., 2020)
	*BnGRF5-L*	Canola	Promoted callus production	None reported	PcUbi4-2	(Kong et al., 2020)
	*ZmGRF5-L1/L2*	Maize	Increased transformation efficiency ~3×	None reported	BdEF1	(Kong et al., 2020)
	*AtWUS*	*Picea glauca*	Involved in somatic embryogenesis and somatic seedling growth	None reported	CaMV 35S	(Klimaszewska et al., 2010)
	*AtWUS*/*WUS*-P2A-*BBM*	*Antirrhinum majus* (Snapdragon)	Induction of shoot regeneration and transformation efficiency	Not specified	CaMV 35S	(Lian et al., 2022)
	*PLT5*	Bok choy and cabbage	Improved shoot regeneration and transformation	Some morphological alterations; viable seed production	CaMV 35S	(Lian et al., 2022)
	*PLT5*	Sweet pepper	Promoted formation of transgenic callus and somatic embryos	None reported	CaMV 35S	(Lian et al., 2022)
	*AtWOX11*/*WOX12*	*Arabidopsis thaliana*	Induction of *de novo* root organogenesis	may impair regeneration	35S and pER8	(Liu et al., 2014)
	*TaDOF5.6* and *TaDOF3.4*	*Triticum aestivum* (Fielder, JM22, Kenong 199)	Up to 2.12X increase in regeneration efficiency	None reported	maize ubiquitin	(Liu et al., 2023b)
	*rZmGOLDEN2*	Zea mays (B104)	1.75X improvement in transformation	None reported	maize ubiquitin	(Luo et al., 2023)
	*rZmGOLDEN2*	*Oryza sativa* (HuaZhan, HuaGuang, DongJing, ZhongJia 8)	1.31–3.44× improvement in transformation	None reported	Rice callus-specific promoter (CSPpro)	(Luo et al., 2023)
	*SlSPR9*	*Solanum lycopersicum* (wild tomato)	6 to 12X increase in transformation efficiency	None reported	Native gene promoters PRP and SlWIND1	(Yang et al., 2024)
	*GmREF1*	*Glycine max* (Dongnong-50)	2 to 5X increase in transformation efficiency	None reported	Native gene promoters PRP and SlWIND1	Yang et al., 2024
	*TaREF1*	*Triticum aestivum* (JM22)	4X increase in transformation efficiency	None reported	Native gene promoters PRP and SlWIND1	Yang et al., 2024
	*ZmREF1*	*Zea mays* (B104)	4X increase in transformation efficiency	None reported	Native gene promoters PRP and SlWIND1	Yang et al., 2024
	*TaLAX1*	Triticum aestivum (Fielder, Chinese Spring)	Significant improvement in transformation	Not reported	ZmUbi	(Yu et al., 2024)
	*MtWOX9-1*	*Medicago truncatula*	Involved in somatic embryogenesis	Not specified	35S	(Tvorogova et al., 2019)
	*PaWOX8/9*	*Picea abies*	Expressed during early somatic embryogenesis	Not specified	35S and XVE	(Zhu et al., 2014a)
	*PaWOX2*	*Picea abies*	Expressed during early somatic embryogenesis	Not specified	35S and XVE	(Zhu et al., 2016)
	*PpWOX2*	*Pinus pinaster*	Change in number and quality of cotyledon embryos	Not specified	35S	(Hassani et al., 2022)
	*PpWOX2*	*Arabidopsis thaliana*	Promote somatic embryogenesis and organogenesis	Not specified	35S	(Hassani et al., 2022)
	*TaWOX5*	*Triticum aestivum* *Maize* *Barley*	Improves transformation efficiency	Not specified	*ZmUbi*	(Wang et al., 2022b)
	*AtGRF5*, *TaGRF4-OsGIF1, ZmWUS* and *ZmBBM*	*Citrullus lanatus*	High transformation efficiency (>20%)	No obvious negative effects on plant growth	*AtUBQ10*	(Pan et al., 2022a)
	*ClGRF4–GIF1* (miR396-resistant)	Watermelon	High transformation efficiency (67.27%),	None reported	CaMV 35S	(Feng et al., 2021)
	*CsGRF3-GIF1*	Cannabis (Hemp)	Improved regeneration efficiency	Genotype-specific (DMG278 had highest regeneration)	CaMV 35S	(Zhang et al., 2021b)
	*TaGRF4-GIF1-ZmBBM*	*Zea mays Hi-II* and *B104*	Enhance regeneration efficiency	No obvious developmental defects	ZmUBI and pPLTP	(Chen et al., 2022a)
	*GRF4-GIF*	*Lactuca* spp.	Enhance regeneration and transformation efficiencies	Abnormal development and maturation of vegetative shoots and leaves	CaMV p35S	(Bull et al., 2023)
	*AtLEC1/L1L*	*Arabidopsis thaliana*	Regulates and enhances somatic embryogenesis	Not specified	CaMV 35S	(Kwong et al., 2003)
	*AtLEC1/LEC2/FUS3*	*Arabidopsis thaliana*	Strong promotion of somatic embryogenesis	Not specified	CaMV 35S	(Horstman et al., 2017)
	*AtLEC1/LEC2*	*Nicotiana tabacum*	Promotes somatic embryogenesis	Not specified	CaMV 35S	(Guo et al., 2013)
	*AtLEC2*	*Arabidopsis thaliana*	Promotes embryo development	Mutant embryos from all lines had abnormal suspensors	CaMV 35S	(Stone et al., 2001)
	*AtLEC2*	*Arabidopsis thaliana*	Induces somatic embryogenesis from asexual cells	Suspensor abnormalities in *lec1–2 fus3–3* double mutants	CaMV 35S	(Lotan et al., 1998)
	*AtLEC2*	*Brassica napus*	Enables somatic embryo formation in cotyledon explants	Not specified	CaMV 35S	(Belide et al., 2013)
	*AtLEC1/LEC2/FUS3*	*Arabidopsis thaliana*	Promotes somatic embryogenesis	Mutants had leafy cotyledons with trichomes on the adaxial surface; defects in suspensor morphology during early embryogenesis; defective seeds (desiccation-intolerant) which had to be rescued before seed maturation.	CaMV 35S	(Gaj et al., 2005)
	*CsFUS3*	*Citrus sinensis*	Promotes somatic embryogenesis	No pleiotropy: T2 transgenic lines developed into fertile plants	35S	(Liu et al., 2018)
	*CsL1L*	*Citrus sinensis*	Transit cells from vegetative to embryogenic phase	Not specified	35S	(Zhu et al., 2014b)
	*DcLEC1*	*Daucus carota*	Induces zygotic and somatic embryogenesis	Embryogenesis in the *lec1, lec2* and *fus3* mutants was abnormal in morphology and the seed maturation process	*AtLEC1*pro	(Yazawa et al., 2004)
	*TcL1L*	*Theobroma cacao* *Arabidopsis*	Expressed in zygotic/somatic embryogenesis	Twisted seeds or a partially developed root apex with normal cotyledons	35SCaMV	(Alemanno et al., 2008)
	*AtSERK1*	*Arabidopsis thaliana*	3- to 4-fold increase in efficiency for somatic embryogenesis	No altered plant phenotype	35S	(Hecht et al., 2001)
	*AtWIND1*	*Arabidopsis, Brassica napus, Solanum lycopersicum, Nicotiana tabacum*	Induces callus without exogenous hormones	May require optimization for shoot regeneration	35S and chemical-inducible XVE	(Iwase et al., 2015)
	*ZmGRF1-GIF*	Maize (B104)	3.5–6.5× improved transformation frequency; efficient CRISPR editing; no impact on plant development	Maize (B104)	ZmUbi	(Vandeputte et al., 2024)
	*AtWIND1*	*Brassica napus* (rapeseed)	Promotes wound-induced *de novo* shoot formation	Did not validate in monocots	35S, XVE, ProWIND1	(Iwase et al., 2015)
	*AtLBD16*, *LBD17*, *LBD18* and *LBD29*	*Arabidopsis thaliana*	Induces callus without exogenous auxin	Validated only in *Arabidopsis*	CaMV 35S; XVE	(Fan et al., 2012)

To mitigate these challenges, researchers have resorted to expressing the MRs only during early growth stages during which they are necessary then restricting their expression in later reproductive stages or entirely eliminating them. One such strategy is the use of tissue specific promoters; for instance, Lowe et al. (2018) regenerated perfectly normal and fertile transgenic maize plants by driving BBM under the maize Phospholipid Transfer Protein (PLTP) promoter and *WUS* under the auxin-inducible Axig1 promoter. Notably, PLTP is only active in dividing embryogenic cells such as in an immature embryo callus while Axig1 is only active under high auxin levels such as during the callus phase (Chu et al., 2019; Lowe et al., 2018).

Another strategy for spatial/temporal control via promoters involves the use of developmentally regulated promoters such as *Oleosin*, *Glb1*, *End2*, and *Ltp2* to drive *Cre* (inducible site-specific recombinase) enabled excision of morphogenic genes in early embryo development. In the study by Wang et al. (2020a), this strategy produced excised events at a rate of 25–100%. The same study also reported a different strategy utilizing an excision-activated selectable marker that produced excised events at a rate of 53–68%. Similarly (Mookkan et al., 2017), demonstrated desiccation induction of *RAB*17_M_: *CRE* expression to remove the *WUS2* and *BBM* transcriptional genes from the transgenic embryogenic callus before transfer to the regeneration medium. The ideal promoter drives enough expression during the regeneration window, but little to no expression in later plant development (**Table 2**).

**Table 2:** Strategies for controlled expression and delivery of morphogenic regulators to enhance plant transformation and regeneration.

Strategy	Mechanism	Promoter(s)	Delivery method	Morphogenic regulators	Crop(s)	Key advantages	Limitations/considerations	References
Tissue-specific expression	MRs driven by egg cell-specific promoter	EC1	*Agrobacterium tumefaciens* C58C1 pMP90	*BnBBM1* and *AtBBM*	*Brassica napus*, *Tomato*, *Arabidopsis*	Enables haploid induction and regulates endosperm proliferation and cellularization.	No phenotypic abnormalities or pleiotropic effects reported	(Chen et al., 2022b)
	MRs under egg cell-specific promoter	PsASGR	Biolistic particle delivery	*PsASGR-BBM*	*Pennisetum glaucum* (Pearl millet)	Induction of parthenogenesis	No adverse developmental effects; main limitation was technical, I.e., ensuring effective expression for inducing parthenogenesis.	(Conner et al., 2015)
	Seed specific	Oleosinpro: WUS 2	Particle bombardment and *Agrobacterium* strain LBA4404	*BBM1* and *WUS2*	*Zea mays*	Involved in somatic embryogenesis and stimulating somatic embryogenesis	Chimeric and often necrotic callus which regenerated only non-transformed plants. Aberrant phenotypes, including thick, short roots	(Lowe et al., 2016)
	Tissue-specific BBM expression	Zm-PLTPpro:: BBM and Nospro:: WUS 2	*A. tumefaciens* strain LBA4404 THY- containing pVir9	*BBM1* and *WUS2*	*Zea mays* inbreds B73 and Mo17	Rapid somatic embryo formation, reduced pleiotropic effects on T0 plants	Germination problems in T1 seed (Due to WUS 2)	(Lowe et al., 2018a)
	Tissue-specific BBM expression, Auxin inducible WUS expression	PLTPpro : HvBBM, Axig1pro:HvWUS	*Agrobacterium tumefaciens* strain EHA105	*HvBBM* and *HvWUS*	Hordeum vulgare (Barley)	3× improvement in transformation efficiency; functional transcriptomic support linking auxin, meristem genes, and epigenetic regulators to SE	Requires immature embryos; genotype dependency may persist; overexpression risks need optimization	(Suo et al., 2021)
Inducible expression	Heat shock induced DNA excision	CaMV 35S + HSP18.2 promoter (FRT/FLP excision system)	*Agrobacterium tumefaciens*-mediated transformation, strain LBA4404	*PaWOX8/9*	*Picea abies*	Expressed during the early stages of somatic embryogenesis	Normal plants with no pleiotropic effects after T-DNA excision	(Deng et al., 2009)
	Drought inducible DNA excision	(1) RAB17M: CRE lox P sites; (2) NOS At : WUS2; (3) UBI M:BBM; (4) UBI M: GFP	*Agrobacterium* AGL1 or EHA101	*PaWOX2*	*Picea abies*	Expressed during the early stages of somatic embryogenesis	Before excision, ectopic expression manifesting as secondary clusters of somatic embryos, folded leaves, and very short plantlets with thick abnormal root structures. Normal regeneration after excision	(Mookkan et al., 2017)
	Drought inducible DNA excision	(i) Zm-Rab17pro::Cre, (ii) NOSpro:: WUS 2, (iii) Zm-Ubipro:: BBM, and (iv) Zm-Ubipro::GFP	*Agrobacterium tumefaciens* strain AGL1	*PpWOX2*	*Pinus pinaster*	Change the number and quality of embryos in cotyledon	Not observed	(Nelson-Vasilchik et al., 2018)
	Desiccation-induced excision	Ubi pro:Bbm, nos pro: WUS 2	*Agrobacterium* strain AGL1 in sugarcane and LBA4404 in rice and sorghum	*PpWOX2*	*Zea mays*	Promote somatic embryogenesis and organogenesis	Not observed after excision	(Lowe et al., 2016)
	Heat-induced excision	Zm-Hsp17.7pro:cre, Axig1pro: WUS 2 and Pltppro : Zm- BBM	*Agrobacterium* strain LBA4404(Thy-), accessory plasmid PHP71539	*TaWOX5*	*Zea mays*	Improve the transformation efficiency	None reported	(Masters et al., 2020)
	Chemical inducible promoter	maize IN2 promoter; WUS	Particle gun delivery	*VvWOX2/VvWOX9*	*Vitis vinifera*	Expressed during the early stages of somatic embryogenesis	Not specified	(Svitashev et al., 2016)
	GR inducible BBM	GR fusion gene, 35S::BBM	*Agrobacterium tumefaciens* GV3101	*VvWOX3/VvWOX11*	*Vitis vinifera*	Expresses at specific stages of somatic embryos (torpedo and cotyledonary)	Reduced pleiotrophic effects	(Srinivasan et al., 2006)
	DEX-inducible	GR fusion gene	*Agrobacterium tumefaciens*	*AtLEC2_GR + AtIPT7/9*	*Nicotiana tabacum*	2–3.5-fold increase in shoot regeneration on hormone-free medium; high-quality embryogenic callus formed under DEX	Variation in lengths of hypocotyls and primary roots	(Li et al., 2019)
	GR-based inducible system	GR fusion	*Agrobacterium tumefaciens*	*WUSCHEL*	*Arabidopsis* *thaliana*	*de novo* embryo formation	Morphologically normal mutant plants by inducer withdrawal	(Zuo et al., 2002)
	GR-based inducible system	GR fusion	*Agrobacterium tumefaciens*	*TcLEC2*	*Theobroma cacao*	Induces somatic embryogenesis in leaves	*TcLEC2-GR* leaf embryos were smaller throughout development	(Fister et al., 2018; Shires et al., 2017)
	Auxin inducible WUS	Zm-PLTPpro:: BBM and Zm-Axig1pro:: WUS 2	*A. tumefaciens* strain LBA4404 THY- containing pVir9	*BBM1* and *WUS2*	*Zea mays*	*De novo* somatic embryo development, callus-free transformation in maize immature embryos, fertile T0 plants, normal plants were recovered without excision	No deleterious pleiotropic effects	(Lowe et al., 2016)
	Chemical inducible promoter	XVE (estradiol-inducible)	Stable transformation via floral dip	*AtWUS*	*Arabidopsis thaliana*	Induces high-frequency somatic embryo formation without auxin or 2, 4-D; direct germination into fertile plants	Requires chemical inducer (estradiol); studied primarily in *Arabidopsis*	(Zuo et al., 2002)
	Chemical inducible promoter	XVE (estradiol-inducible)	*Agrobacterium*-mediated	*AtWUS*	*Nicotiana tabacum*	Enables regeneration from root tip; applicable to recalcitrant species	Requires β-estradiol; shoot formation from root segments needs cytokinin	(Rashid et al., 2007)
	Estradiol-inducible overexpression of morphogenic regulators	Estradiol-inducible (XVE)	*Agrobacterium*-mediated	*AtWOX2/WOX8/WOX9*	*Nicotiana tabacum*	Promote regeneration from leaf segments and free cells	Not specified	(Kyo et al., 2018)
	Estradiol-inducible overexpression of morphogenic regulators	Estradiol-inducible (XVE)	*Agrobacterium*-mediated	*AtWUS* and *CHAP3A* (*PmLEC1*)	*Picea glauca* (white spruce)	Demonstrated transgene expression throughout somatic seedlings	Developmental disruption of embryos; inhibited germination; no SE induction despite high expression	(Klimaszewska et al., 2010)
	Jasmonate-induced *AtWUS* expression triggered callogenesis and embryogenesis without exogenous hormones	*vsp1* (jasmonate-inducible)	*Agrobacterium*-mediated (including hairy root transformation)	*AtWUS*	*Medicago truncatula*	Enabled PGR-free callogenesis and somatic embryogenesis in leaf and root explants	Effectiveness depends on jasmonate responsiveness in tissue	(Kadri et al., 2021)
Altruistic transformation	Herbicide (GOI); No selection for MRs	PLTP:: WUS 2, with 3× viral enhancer::UBI:: BBM	*Agrobacterium* (dual T-DNA)	*ZmWUS2* and*ZmBBM1*	*Zea mays*	BBM and WUS supplied in trans; only the target gene cassette was integrated		(Hoerster et al., 2020)
	GOI marker only	Pltp and 3×Enh–UBI for WUS 2/ BBM	Biolistics	*ZmWUS2* and*ZmBBM*	*Zea mays*	Transient MR expression boosts genome editing without MR integration		(Chen et al., 2022a)
	Marker for GOI; No marker for MRs	Pltp and 3×Enh–UBI for WUS 2/ BBM	cobombardment and *Agrobacterium tumefaciens* transformation	*ZmWUS2* and *ZmBBM*	*Zea mays*, *Oryza sativa*, *Sorghum bicolor* and *Saccharum officinarum*	Co-delivered but not selected; transient expression sufficient for regeneration		(Lowe et al., 2016)
	Dual-vector transformation with differential selection	Pltp and 3×Enh–UBI for WUS 2/ BBM	*Agrobacterium* (dual vector)	*ZmWUS2* and *ZmBBM*	*Zea mays*	MRs carried on a separate T-DNA with a different selection marker (hygromycin), while the gene of interest was selected with phosphinothricin (Bialaphos); resulting transformants lacked the MR construct		(Kang et al., 2023)
	Altruistic delivery of viral and conventional T-DNA vectors	OsEf1a: *GRF4-GIF1*; ZmUbi: *WUS2*; 3xEnh-ZmUbi: *BBM*	Foxtail Mosaic Virus (FoMV) and *Agrobacterium*	*ZmBBM*, *ZmWUS2*, *ZmWox2a* and TaGRF4-TaGIF1	*Sorghum bicolor* leaf explants	Embryo formation improved with altruistic GRF4-GIF1 delivery (61.3%) compared to regular methods (43–50%). Using viral vectors with GRF4-GIF1 or BBM / WUS 2 boosted this further to over 75%	FoMV expression is transient, with viral transcripts disappearing within two weeks post-inoculation	(Butler et al., 2025)
Excision system + Altruistic transformation	CRE/loxP-based removal of morphogenes and marker after regeneration	PLTP (WUS 2), ZmUbi (BBM), GLB1 (CRE), 35S (CRE)	*Agrobacterium*-mediated (MAT and Altruistic dual-strain)	*ZmWUS2* and *ZmBBM*	*Sorghum bicolor*	Reduced transformation time by ~50%- Enabled transformation of recalcitrant lines- Editing of *PDS* demonstrated- Clean events without morphogenes via excision or altruism	Requires co-infection strategy; transgene-free lines must be screened; not all genotypes may respond equally well	(Aregawi et al., 2022)

### 5.2 Transient expression of MRs for genetic transformation and genome editing

Ensuring that MRs do not express stably in the host genome offers another popular and effective way of sidestepping their lasting drawbacks. As such, the MRs only act during the early transformation stages but are absent in the transgenic plant. Some of the methods proven to aid in this transient set up include altruistic transformation, use of viral vectors, in planta transformation, plasmid-based transient expression, and direct delivery as a protein.

Among these, co-culture with a morphogene vector that doesn’t integrate (altruistic transformation) stands as a favorite in monocots. This involves co-delivering the construct that harbors the gene of interest (GOI) with another that carries a morphogenic construct but has a different selectable marker. Previously, a method using a non-integrating *WUS2* gene approach recovered fertile T0 plants free of morphogenic genes (Hoerster et al., 2020; Syombua et al., 2024). The studies demonstrated that co-infecting with two *Agrobacterium strains*, one with a *WUS2* expression cassette, and the other with a combination of both selectable and visual marker cassettes, produced transformed T0 plants that contained only a single copy of the selectable marker T-DNA, without the integration of *WUS2*. However, it is important to optimize the process to determine the optimal ratio of the two *Agrobacterium* strains that enables modest recovery of selectable marker-containing T0 plants that do not contain the morphogene (Kang et al., 2023).

### 5.3 Use of MRs for *de novo* meristem induction

While conventional transformation methods target pre-existing shoot apical meristems (SAMs) and axillary buds (Zhang et al., 2017b), recent breakthroughs have demonstrated that MRs can transiently induce the formation of new meristematic tissues directly from somatic cells (**Table 3**). First demonstrated by (Maher et al., 2020), this novel strategy involves co-delivering MRs, in this case *WUSCHEL2 (WUS2)*, *SHOOT MERISTEMLESS* (STM), and the cytokinin biosynthesis gene *ISOPENTENYL TRANSFERASE* (*IPT*), along with genome editing reagents into somatic cells. The transient expression of these factors in *Nicotiana benthamiana* promoted the formation of new meristems at the infection sites and genome-edited shoots were subsequently regenerated. Notably, these edits were stably transmitted to the next generation (Maher et al., 2020).

**Table 3:** Emerging strategies for delivering morphogenic regulators and gene editing tools into plant cells, including viral vector delivery, *de novo* meristem induction, and CRISPR-based gene activation.

Strategy	Mechanism	Promoter(s)	Delivery method	Morphogenic regulators	Crop(s)	Key advantages	Limitations/considerations	References
Viral vector delivery								
	Transient MR expression	FoMV replication signals (native viral promoters)	Viral vector (FoMV) via rub-inoculation	*BBM*, *WUS2*, *Wox2a*, *GRF4-GIF1*	Sorghum bicolor	High embryogenic callus formation (79–75%) without genomic integration	Transient expression only; stability of long-term expression unknown	(Maher et al., 2020)
	Transient MR expression	FoMV native promoters	Viral vector (FoMV)	*BBM*, *WUS2*, *Wox2a*, *GRF4-GIF1*	Multiple Poaceae (e.g., maize, sorghum)	Broad applicability via leaf transformation; avoids *Agrobacterium* integration	Need follow-up on fertility and field performance	Maher et al., 2020
	Altruistic delivery of viral and conventional T-DNA vectors	OsEf1a: *GRF4-GIF1*; ZmUbi: *WUS2*; ZmUbi (3× enhancer): *BBM*	Foxtail Mosaic Virus (FoMV) and *Agrobacterium*	maize Zm*BBM*, Zm*WUS2*, *ZmWox2a* and TaGRF4-TaGIF1	*Sorghum bicolor* leaf explants	Embryo formation improved with altruistic GRF4-GIF1 delivery (61.3%) compared to regular methods (43–50%). Using viral vectors with GRF4-GIF1 or BBM / WUS 2 boosted this further to over 75%	FoMV expression is transient, with viral transcripts disappearing within two weeks post-inoculation	(Butler et al., 2025)
*De novo* meristem induction	In planta delivery of MRs	Ubiquitin / constitutive T-DNA DR + CRISPR cassette	*Agrobacterium* injection or vacuum infiltration	*WUS2* + *STM*, *BBM*	Tomato, Potato, Grape, other dicots	Tissue culture bypass; regenerated edited plants transmitted to next generation	Potential somatic mosaicism; limited monocot examples	Maher et al., 2020
		CaMV 35S	*Agrobacterium* injection into stem tissue	*PLT5*	Snapdragon	Promoted callus and shoot regeneration at wound sites	Some morphological alterations; stable inheritance confirmed	Lian et al., 2022
		CaMV 35S	*Agrobacterium* injection into stem tissue	*PLT5*	Tomato	Highest transformation efficiency among tested MRs	Not reported	Lian et al., 2022
		CaMV 35S	*Agrobacterium* injection into stem tissue	*WIND1*	Snapdragon	Improved transformation efficiency	Not specified	Lian et al., 2022
		CaMV 35S	*Agrobacterium* injection into stem tissue	*WUS*	Snapdragon	Increased transformation potential	Not specified	Lian et al., 2022
		CaMV 35S	*Agrobacterium* injection into stem tissue	*WUS-P2A-BBM*	Snapdragon	Promoted shoot regeneration at wound sites	Morphological alterations; pleiotropic effects	Lian et al., 2022
		Synthetic activator (ARR12, WUS driven)	*Agrobacterium* leaf infiltration with morphogenic cocktail	*WUS* and *ARR12*	Arabidopsis	Precise control over shoot regeneration	Synthetic system requires optimization	Zhang et al., 2017a
CRISPR-Combo								
	Co-delivery of morphogenic factors and CRISPR reagents in one T-DNA cassette, for dCas9-based activation of *OsWOX11*	U6 (for sgRNA), 35S/Ubi (for Cas9)	*Agrobacterium*-mediated transformation	Genome editing (e.g., *ALS*, *OsPDS*) + Activation (e.g., *FT*, *OsWOX11*)	*Arabidopsis thaliana*, *Oryza sativa* (rice)	Combines DSB or base editing and gene activation in one construct; enables speed breeding and hormone-free regeneration	Requires precise sgRNA engineering; efficiency varies between constructs/crops	(Pan et al., 2022b)

The broad applicability of this method has since been demonstrated though its successful application in tomato, potato, grape, two brassica cabbage varieties (*Brassica rapa* var. Bok choy and Pei‐Tsai) and citrus, among other crops (Kelly et al., 2025; Lian et al., 2022). Lian et al. showed that *PLETHORA5* (*PLT5*), *WOUND INDUCED DEDIFFERENTIATION 1* (*WIND1*), and *WUS* significantly enhanced the efficiency of *in planta* transformation. The study further reported that *PLT5* induces callus formation and shoot regeneration at wound sites on aerial tissues, enables stable transgene inheritance in tomato and cabbage progeny and facilitates *in vitro* somatic embryogenesis in sweet pepper (Lian et al., 2022). A more recent report is the IPGEC (in planta genome editing in citrus) system (Kelly et al., 2025), an *in planta* approach for genome editing in soil‐grown citrus plants via direct transformation of young seedlings. The *Agrobacterium*-based editing system was designed to transiently co‐deliver three gene modules, including (i) MRs (ii) Cas9 and sgRNAs targeting the carotenoid biosynthetic gene *PHYTOENE DESATURASE* (*PDS*), and (iii) accessory genes to enhance T-DNA delivery. Notably, the integrated system significantly improved *de novo* shoot induction and regeneration efficiency of albino edited tissue (Kelly et al., 2025).

In the agroinfiltration-based Fast-TrACC method (Cody et al., 2023), *Agrobacterium* is briefly applied to intact seedlings or explants to deliver MRs and CRISPR, then the bacteria are washed off. Following a transient co-culture, the plant tissue forms edited shoots, hence bypassing laborious tissue culture processes. Such *In planta* transformation systems and associated *de novo* meristem induction approaches offers a powerful alternative to tissue culture dependent regeneration and facilitates genome engineering even in recalcitrant species (Zhang et al., 2017c).

### 5.4 Use of inducible promoter systems to fine-tune expression

Inducible expression systems that remain off until the availability of a specific signal allow temporal control over the trade-off between high-level transgene expression and its pleiotropic effects on plant growth. For example, chemical, heat shock, or light inducers can be activated at specific developmental stages to initiate regeneration and then switched off to allow normal plant development (Jiang et al., 2024b). This strategy minimizes off-target effects and developmental defects. In maize, Wang et al. (2020a) utilized heat shock promoters to drive Cre recombinase expression in transgenic calli; a brief heat pulse was then applied during early embryo development to excise the WUS/BBM genes from the T-DNA. This allowed for regeneration of fertile plants free of the morphogenic transgenes (Wang et al., 2020a).

The XVE chemical-inducible system is another common inducible promoter system; it uses a chimeric transcription factor activated in the presence of the estrogen analog β-estradiol. Upon activation, it drives GOI expression under the LexA operator promoter. In cotton, for instance, placing *GhLBD18* (*LATERAL ORGAN BOUNDARIES DOMAIN 18*) transcription factor under the XVE inducible promoter (pER8) ensured that callus proliferation only occurred when estradiol was added to the medium. Similarly, WUS could be placed under a dexamethasone-inducible promoter then apply dex only for a short duration to spark regeneration (Omelina et al., 2022; Xinying et al., 2024). Another approach is light-inducible systems, such as using a plant usable light-switch elements (PULSE) or a UVresponsive promoter to allow spatial control by illuminating only part of a tissue to induce local organ formation (Ochoa-Fernandez et al., 2020).

### 5.5 Transient activation of MRs via CRISPR and RNA guides

Rather than introducing DNA cassettes encoding MRs, RNA-based approaches deliver synthetic RNA molecules to influence morphogenic pathways. Thus, this RNA-guided approach does not introduce any new coding sequences but achieves MR overexpression by turbocharging the plant’s existing morphogenic genes (**Table 3**). For instance, a recent preprint (Butler et al., 2025) developed an inducible CRISPR activation system where multiplexed guide RNAs were used to turn on native morphogenic genes *in planta*. Instead of adding an extra WUS gene, the system employs a dCas9 activator and guide RNA to turn on the plant’s endogenous WUS gene. Notably, the study reports regeneration of fluorescent shoots from both conventional and viral vectors, providing evidence on the broad delivery strategies of RNA-based MRs for improving plant transformation (Butler et al., 2025). Similarly, a previous study used a chemically inducible CRISPR activation tool to activate endogenous BBM and LEC2, and achieved enhanced regeneration efficiencies in alfalfa, woodland strawberry, and sheepgrass (Zhang et al., 2024c).

A CRISPR-Combo approach was recently shown to aid the editing of one gene and simultaneous activation of another in the same cell using a dual gRNA system (Pan et al., 2022b). The system incorporates catalytically active Cas9 and transcriptional activation domains to allow one gRNA to direct editing of the GOI and the other upregulates gene transcription by targeting the promoter region of a different gene. Pan et al. first used CRISPR-Combo to induce early flowering in Arabidopsis by editing trait genes and simultaneously activating the flowering gene FT. The approach was then extended to rice and poplar, where editing of a GOI and simultaneous transient activation of the endogenous WOX11 resulted in higher regeneration efficiencies. Since the activation component depends solely on gRNAs and no transgene integration, it mitigated the risk of pleiotropic effects and enabled the recovery of edited, transgene-free plants in subsequent generations (Pan et al., 2022b).

## 6 Challenges and limitations

Developmental defects: The constitutive expression of some morphogenes causes aberrant phenotypes, such as organ malformation, fasciation, and sterility (Lowe et al., 2016). As such, the need to silence the genes’ activity post regeneration is imperative. Current mitigation strategies include the use of inducible promoters such as estrogen or heat shock inducible and excision mechanisms such as *Cre/lox* from bacteriophage P1, *Flp/frt* from *Saccharomyces cerevisiae*, R/RS from *Zygosaccharomyces rouxii*, and *Gin*/*gix* from bacteriophage (Gordon-Kamm et al., 2019; Wang et al., 2020b). Although no apparent reproductive or morphological aberrations were observed in regenerated maize with excised WUS/BBM, there is need for downstream phenotypic and genetic analysis of progeny for subtle anomalies. Could transient morphogene expression have caused epigenetic reprogramming, stress induced mutations, or hidden somaclonal variation? Besides, current excision mechanisms do not guarantee complete removal of MRs and the efficacy differs across genotypes and species (Hoerster et al., 2020).

Licensing and Intellectual property (IP) constraints: The usage of key morphogenic regulators such as BBM/WUS, GRF/GIF and excision constructs are patented by major agbiotech companies or institutions (Gordon-Kamm et al., 2014; Jorge et al., 2021; Stern and Hynes, 2015). Although these technologies are free for use by academic institutions under research use exemptions, entities using them to develop seed systems or for commercialization need to obtain appropriate licensing and freedom to operate agreements. Notably, such systems could constraint accessibility for researchers in low resource settings and breeding programs in the public sector (Van Overwalle et al., 2006).

Species and genotype dependency of response to MRs: Plant response to MRs varies across species and genotypes, and some species require tailored MRs combinations (Wang et al., 2025). Besides, morphogenic regulators alone do not solve issues like recalcitrance to *Agrobacterium* infection or extreme sensitivity of tissues to wounding, hence parallel fine tuning of other parameters like *Agrobacterium* strain, explant type, media composition and culture conditions are needed for each case (Duan et al., 2022; Wang et al., 2023).

Regulatory hurdles and public acceptance issues: Heterologous usage of MR genes, especially in vegetatively propagated crops where transgene segregation is challenging, could add an additional layer of regulatory scrutiny during commercial deployment. Such concerns could also further complicate public acceptance of such products.

## 7 Recent advances and future prospects

The field of plant transformation is currently in a dynamic period thanks to the advent of morphogenic genes and breakthroughs in developmental biology. Among these is the recent discovery of the peptide regenerating factor REF1, as it provides a completely novel mode for enhancing plant regeneration. Unlike conventional MRs that are typically incorporated in a gene construct then transformed into the plant, REF1 is introduced into cells by externally treating plant tissue with the peptide signal (Yang et al., 2024). Notably, REF1’s broad effectiveness across species suggests it could become a standard supplement in transformation protocols to boost shoot formation. Besides, its discovery suggests there is potential to identify or engineer other peptides with similar effects (Youngstrom et al., 2025).

In a recent study, Zhang et al. (2024c) used an inducible CRISPR activation libraries and multiplexed guide RNAs to upregulate gene combinations in Arabidopsis. The study subsequently identified pairs with synergistic effects on regeneration. This high-throughput approach offers unprecedented ways to systematically pinpoint which gene sets promote best regeneration in recalcitrant species. As such, we can now apply CRISPR activation to map out regeneration gene networks for different crops.

The possibility of using MR for *in planta* Transformation will offer the long-awaited transition from tissue culture-based plant engineering systems. Tissue culture-free transformation systems have great potential in addressing genotype-dependency challenges, shortening transformation timeline, and improving operational efficiency by significantly reducing personnel and supply costs (Zhong et al., 2025). Recent advances inch toward that: for example (Maher et al., 2020), demonstrated the induction of *de novo* meristems directly from somatic tissues, bypassing the need for traditional callus-based regeneration. In *Arabidopsis*, co-delivery of *WUS/STM* enabled shoot formation from leaf and stem tissues, significantly accelerating transformation workflows (Maher et al., 2020). Similar strategies have been extended to woody species like citrus, where transient expression of *AtWUS* enabled editing of the PDS gene via the *in planta* IPGEC approach (Kelly et al., 2025). In another study, soil-grown tomato plants and snapdragon (*Antirrhinum majus*) were successfully transformed by inducing meristematic outgrowths on wound sites using MRs (Lian et al., 2022).

Integration with speed breeding and haploidy: Morphogenic regulators could accelerate doubled haploid production – an area critical for crop breeding. For instance, maize haploid inducer lines could potentially express a morphogenic factor to cause haploid embryo-like structures to germinate more readily or undergo genome doubling in tissue culture. Similarly, in species where making haploids is tough, adding BBM might coax unfertilized ovules to develop (some BBM variants cause parthenogenesis in the absence of fertilization, which is being looked at for synthetic apomixis). So there’s interplay with creating homozygous lines faster.

## 8 Regulations surrounding genome editing

CRISPR–Cas technology faces challenges such as reagent delivery, precision editing with minimal off-target effects, and the production of transgene-free plants. The integration of CRISPR components like Cas, guide RNA, and selectable markers into the host genome raises regulatory issues. However, advancements in gene-editing, DNA-independent RNP delivery, grafting-mediated editing, nanoparticles, and viral delivery are facilitating transgene-free crop improvement and aligning with biosafety regulations. Further, the transgenic-free delivery method and regeneration protocols enhance efficiency and minimize cytotoxicity, paving the way for broader applications of this DNA-free technology. Regulatory frameworks for genome-edited crops differ worldwide, affecting commercialization, as some countries accept these technologies while others are still debating their legal status (Ramakrishnan et al., 2025). In most countries, genome editing products are regulated according to product- and process-based guidelines (Fernández Ríos et al., 2025). Guidelines based on processes emphasize recombinant DNA technology, while product-based rules evaluate organisms based on their attributes, regardless of the method of development. DNA delivery methods such as *Agrobacterium*-mediated transformation and particle bombardment can lead to random transgene integration in plant genomes, potentially disrupting essential genes and causing variability in transgene expression. Consequently, such genetically modified organisms (GMOs) are subject to regulatory scrutiny. To mitigate these issues, DNA-free genome editing technologies have been developed, particularly through the use of pre-assembled ribonucleoproteins (RNPs) comprising Cas proteins and synthesized guide RNAs. This method ensures that no recombinant DNA is involved, rendering the edited plants transgene-free. RNPs demonstrate advantages in precision and transient activity within plant cells, allowing targeted genomic editing while minimizing off-target effects and reducing risks of mosaicism and toxicity, as they are degraded shortly after delivery (Ramakrishnan et al., 2025; Maia et al., 2024). The regeneration of whole plants from edited protoplasts remains a significant challenge for many species employing RNA-mediated genome editing. To address this, alternative strategies such as morphogenic induction have been proposed. These approaches involve the co-expression of morphoregulator alongside gene editing cassettes, enabling the formation of gene-edited shoots and ensuring the heritable transmission of modified DNA to subsequent generations. Current research emphasizes enhancing the delivery, selection, and regeneration of CRISPR reagents across diverse plant species, thereby broadening the application of CRISPR technology in crop improvement and agricultural innovation. The existing regulatory framework for the commercialization of genome editing products is regulated by both product- and process-oriented criteria. The *Agrobacterium*-mediated introduction of CRISPR vector reagents into plant cells and tissues faces regulatory obstacles due to random integration, and the subsequent segregation to remove transgenes is a laborious operation, rendering it unfeasible for trees and vegetative plants. Furthermore, the persistent exposure of genomic DNA to CRISPR agents throughout the editing procedure may lead to off-target mutations and the emergence of chimera mutants (Hashimoto et al., 2016). Additionally, polyploid organisms frequently necessitate numerous gRNAs, hence complicating the transformation of CRISPR vector systems. In light of the aforementioned facts, plant virus-based replicons and ribonucleoprotein-mediated gene delivery, along with integration of the morphogenic regulator-based improved transformation system, are increasingly utilized as alternatives to *Agrobacterium* for the introduction of genome editing tools into plants, aiming to enhance crop improvement while minimizing the time and labor associated with the current regulatory framework.

## 9 Conclusion and future directions

Morphogenic regulators and peptide factors are reshaping the landscape of plant transformation and gene editing. What was once an art of coaxing recalcitrant tissues to regenerate is becoming a predictable, engineerable process. By tapping into the plant’s own developmental control circuits through genes like WUS, BBM, LEC, GRF-GIF, and signals like REF1 peptide, researchers can dramatically improve the efficiency and speed of obtaining transgenic or edited plants across a wide range of species. The successes in major crops like maize and wheat have broken the dogma that some elite genotypes are untransformable. Furthermore, the convergence of morphogenic regulators with cutting-edge genome editing and synthetic biology heralds a new era for plant science and agriculture. The ultimate vision is to establish a robust and streamlined plant transformation system that will allow us to move from a genetic concept to edited plant in a season for any crop of interest. Nonetheless, realization of the full potential of these advances will require continued innovation. For instance, more refined control systems will be needed to ensure MRs do their job and then exit fully, without trace. Inducible and tissue-specific systems are a great start, and the trend will be to make these ever more precise. Considering the rapid progress to date, however, this is a highly attainable goal. The integration of RNA-seq, ATAC-seq, and CUT&Tag techniques enables researchers to elucidate gene regulation and cellular function, facilitating the identification of candidate MRs for understanding transcriptional regulatory networks that govern embryonic callus formation and organogenesis (Ouyang et al., 2021; Liu et al., 2023b). These approaches uncover novel transcription factors and peptides implicated in regeneration, along with transcriptome and epigenetic alterations during callus maturation and shoot regeneration. The integration of artificial intelligence and machine learning, combined with the functional validation of newly identified morphogenic factors, enhances the understanding of somatic embryogenesis, *de novo* regeneration, and addresses challenges associated with plant regeneration, thereby significantly improving the transformation efficiency of numerous economically important crops. Many technology stakeholders are set to benefit from these advances, including scientists being able to accelerate functional genomics research and breeders tailoring crops to the needs of consumers and changing climates. The society is also set to benefit as we adapt our food system to meet global challenges. By expediting plant transformation and gene editing, morphogenic regulators are not just tools of convenience, but also catalysts helping us to engineer crops for a sustainable future.
